# Emerging epidemiological trends of multiple sclerosis among adults aged 20–54 years, 1990–2021, with projections to 2035: a systematic analysis for the global burden of disease study 2021

**DOI:** 10.3389/fneur.2025.1616245

**Published:** 2025-07-10

**Authors:** Ling-yun Wang, Wen-fu Wang, Shu-yin Hui, Li Yang, Yue-xian Liu, Hang-juan Li

**Affiliations:** Department of Neurology, 920th Hospital of the Joint Logistics Support Force, People’s Liberation Army, Kunming, China

**Keywords:** multiple sclerosis, GBD (2021) database, incidence, mortality, DALYs (disability adjusted life year), public health

## Abstract

**Background:**

Over recent decades, clinical, scientific, and public awareness of multiple sclerosis (MS) has increased significantly. This study aims to analyze global trends in the incidence, mortality, and disability-adjusted life years (DALYs) of MS among adults aged 20–54 years from 1990 to 2021.

**Methods:**

Using data from the Global Burden of Disease (GBD) database, this study evaluated MS incidence, mortality, and DALYs in adults aged 20–54 years. Data from 204 countries and territories were stratified by age, sex, and geographical location. Annual percentage changes (APC) were calculated using Joinpoint regression, and estimated annual percentage changes (EAPC) were derived through log-linear regression modeling. Bayesian age-period-cohort (BAPC) modeling was employed to forecast future disease burden.

**Findings:**

Globally, in 2021, there were 51,904.12 incident MS cases (95% UI, 44,998.80–59,073.37), 4,738.38 deaths (95% UI, 4,492.03–5,024.11), and 512,985.58 DALYs (95% UI, 428,133.20–610,308.62). Between 1990 and 2021, global incidence increased by 49.48% (95% UI, 44.34–55.15%), mortality rose by 18.21% (95% UI, 11.60–25.45%), and DALYs increased by 43.22% (95% UI, 36.79–48.94%). The High SDI region reported the highest rates of incidence (4.38 per 100,000; 95% UI, 3.93–4.83), mortality (0.41 per 100,000; 95% UI, 0.39–0.42), and DALYs (45.00 per 100,000; 95% UI, 37.22–53.12). Regionally, Western Europe had the highest number of cases (10,964.14; 95% UI, 9,572.30–12,318.88). At the national level, the United States reported the most MS incident cases in 2021 (9,388.21; 95% UI, 8,469.23–10,275.59). Sweden had the highest national incidence rate at 10.12 per 100,000 (95% UI, 8.69–11.66), with an EAPC of 0.24 (95% CI, 0.10–0.38).

**Interpretation:**

The global incidence, mortality, and DALYs associated with MS have shown increasing trends, although substantial variations persist across different SDI regions. A comprehensive understanding of MS epidemiology is essential for improving global disease prevention and control efforts.

## Introduction

Multiple sclerosis (MS) is a prevalent and disabling autoimmune disorder characterized primarily by two clinical forms (1, 2). In relapsing–remitting MS, patients experience intermittent neurological symptoms with initial remission phases that ultimately progress to permanent disability. Progressive MS, conversely, is marked by continuous deterioration commonly culminating in wheelchair dependence or bedridden states (3). The pathophysiology of MS involves two distinct processes: inflammation and demyelination, alongside astrocytic proliferation (gliosis) and neurodegeneration (4). Pathological damage is confined exclusively to the central nervous system (CNS), sparing the peripheral nervous system (5). Clinical presentations of MS vary widely, influenced by lesion location and severity within the CNS (6). Symptoms can be severe at onset or insidiously develop, remaining undetected for months or even years. Predominantly affecting young adults, MS exhibits both relapsing–remitting and progressive disease trajectories, causing neurological impairments that lead to substantial healthcare expenditures and impaired employment prospects (7).

The epidemiology of MS is well-documented; global estimates suggest an increase from approximately 2.2 million cases in 2016 to 2.8 million in 2020 (8). Region-specific incidence trends illustrate this rise. In southern Norway, incidence increased from 10.2 (2003–2007) to 13.1 (2008–2012) (9). Individuals of Northern European descent and the White ethnic group exhibit a higher disease risk, with prevalence rates demonstrating a decreasing gradient as distance from the equator increases (10). In northeastern Italy, rates climbed significantly from 0.9 (1960–1965) to 6.5 (2011–2015) (11). Similarly, multiple studies from the Middle East have indicated sharp increases in MS incidence (12–14). These epidemiological trends underscore the considerable burden placed upon individuals and families, highlighting MS as a significant global public health issue.

Previous assessments using data from the Global Burden of Disease (GBD) 2019 study have quantified the worldwide burden, temporal trends, and age-period-cohort effects of MS (15). Building on these foundations, the current study aims to elucidate global and regional temporal trends in incidence, mortality, and disability-adjusted life years (DALYs) among adults aged 20–54 years from 1990 to 2021. Additionally, Bayesian age-period-cohort (BAPC) models are employed to forecast the disease burden of MS through 2035. This study seeks to inform global strategies for diagnosis and treatment, with the ultimate goal of mitigating the global impact of MS.

## Methods

### Overview and methodological details

The GBD study, coordinated by the Institute for Health Metrics and Evaluation (IHME) at, University of Washington, provides one of the most comprehensive epidemiological frameworks globally. It quantifies health losses from diseases, injuries, and risk factors, enabling comparative assessments across countries and regions (16). The GBD utilizes three principal metrics: incidence, mortality, and DALYs. DALYs integrate years of life lost due to premature mortality (YLL) and years lived with disability (YLD), calculated as follows:

YLD = Disease prevalence × disability weight.

YLL = Number of deaths × standard life expectancy at age of death.

Disability weights, ranging from 0 (perfect health) to 1 (death), are assigned through expert consensus. Data on MS cases, incidence, mortality, and DALYs among adults aged 20–54 years from 1990 to 2021 were retrieved from the GBD database^1^, covering 204 countries and territories (17). Analyses considered multiple dimensions, including sex, age groups (20–24, 25–29, 30–34, 35–39, 40–44, 45–49, and 50–54 years), and location. Ethnic and racial subgroup analyses were not performed due to data unavailability. This cross-sectional study involved aggregated population-level data without identifiable personal information; thus, informed consent requirements were waived by the 920th Hospital of the Joint Logistics Support Force, People’s Liberation Army Ethics Committee. Reporting adhered strictly to the Strengthening the Reporting of Observational Studies in Epidemiology (STROBE) guidelines (18).

### Sociodemographic index

The sociodemographic index (SDI), ranging from 0 (lowest) to 1 (highest), quantifies regional socioeconomic development based on fertility rates, education levels, and income per capita (19). Previous studies have demonstrated associations between SDI and disease burden (20). In this study, countries and regions were categorized into five SDI groups (low, low-middle, middle, high-middle, and high) to examine the relationship between MS burden and socioeconomic development.

### BAPC model projection to project future MS burden

We employed the BAPC model due to its capability to address the complexity, high dimensionality, and sparsity inherent in large epidemiological datasets such as GBD 2021 (21). The BAPC model extends traditional generalized linear models (GLMs) within a Bayesian framework, dynamically integrating age, period, and cohort effects. These effects are assumed to evolve smoothly over time, employing second-order random walks for precision. A significant advantage of the BAPC approach is its use of integrated nested Laplace approximation (INLA), efficiently circumventing convergence issues associated with Markov Chain Monte Carlo techniques, thus enhancing computational efficiency. The flexibility and robustness of the BAPC model make it particularly suitable for long-term projections of disease burden. Utilizing the R package “BAPC” and demographic forecasts from IHME, we generated detailed predictions accounting for the intricate interactions among age, period, and cohort effects.

### Statistical analysis

The primary indicators of MS burden were incidence, mortality, DALYs, and their corresponding rates per 100,000 population, reported alongside 95% uncertainty intervals (UIs) following GBD protocols. The estimated annual percentage change (EAPC) and its 95% confidence interval (CI), determined via linear regression modeling, quantified temporal trends in MS burden. An increasing trend was indicated when both EAPC and the lower limit of its 95% CI were positive, whereas a decreasing trend was suggested when both values were negative (22). Additionally, annual percent changes (APCs) and their 95% CIs were computed using Joinpoint regression to evaluate internal trends within discrete time segments (23). All analyses were performed using R software (version 4.4.2), with statistical significance defined at *p* < 0.05.

## Results

### Global burden trends

#### Incidence

**Figure 1:**
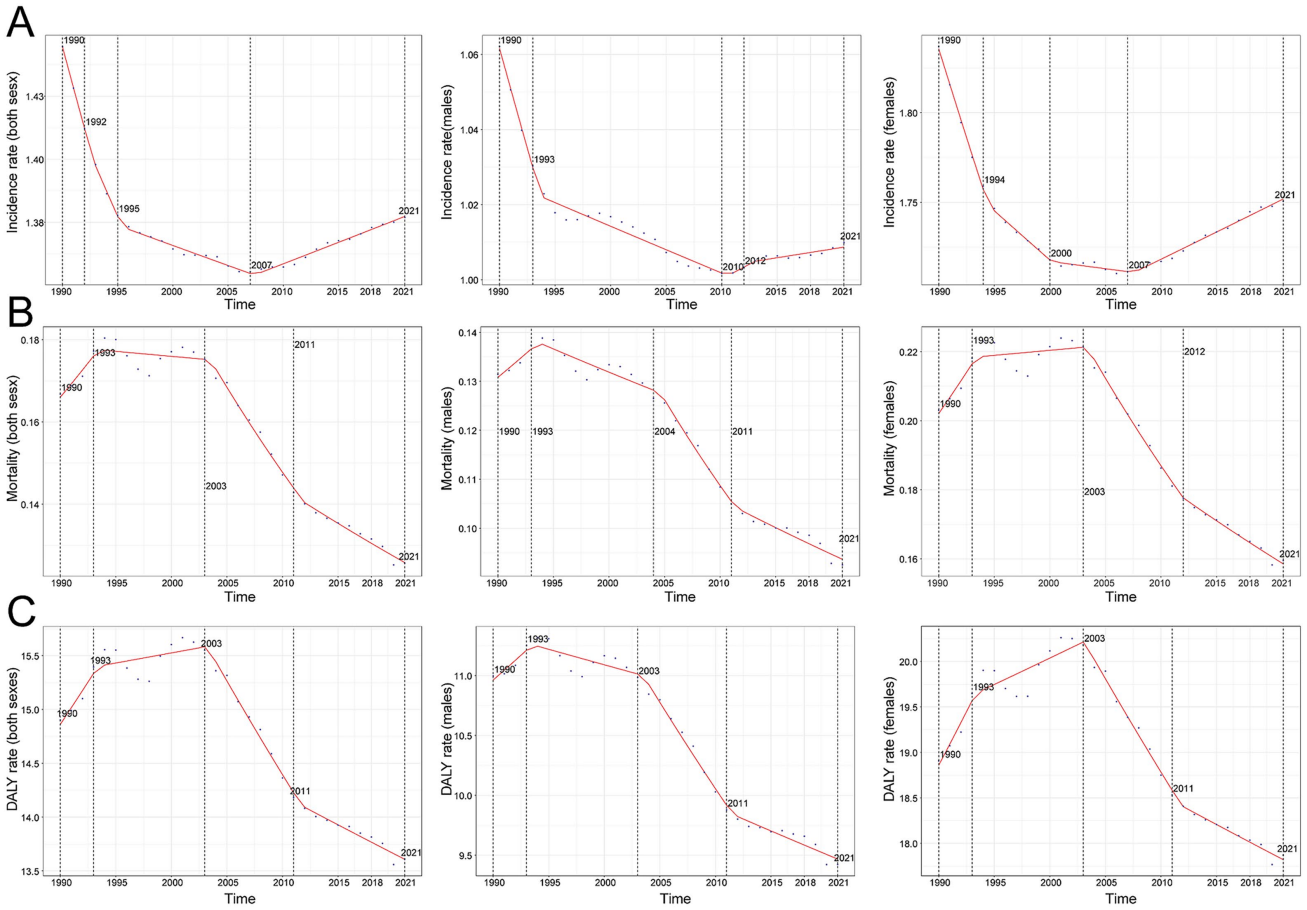
Annual percent change (APC) and trends in global multiple sclerosis incidence, mortality, and disability-adjusted life years (DALYs) from 1990 to 2021. **(A)** Incidence rate. **(B)** Mortality. **(C)** DALY rate.

**Figure 2:**
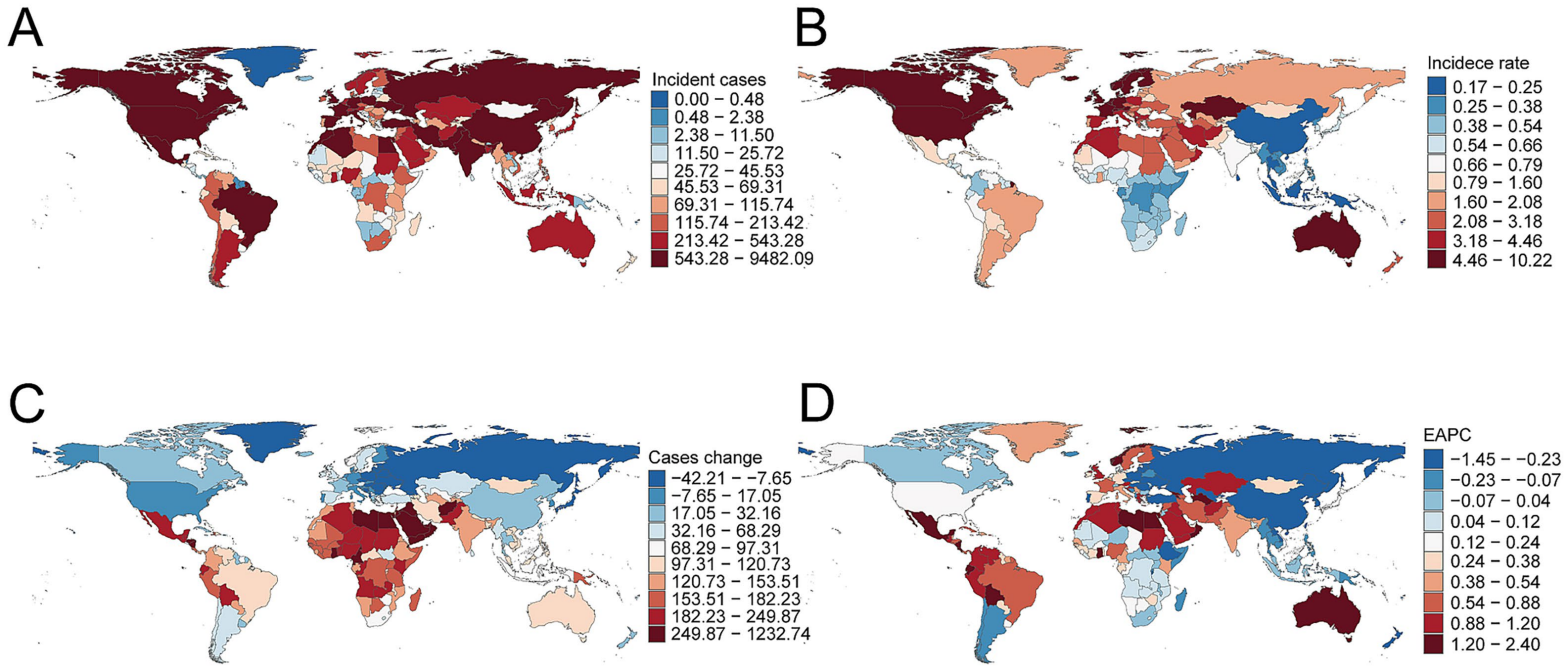
Incidence of multiple sclerosis across 204 countries and territories. **(A)** Number of incident cases. **(B)** Incidence rate. **(C)** Change in incident cases. **(D)** Estimated annual percentage change in incidence.

**Figure 3:**
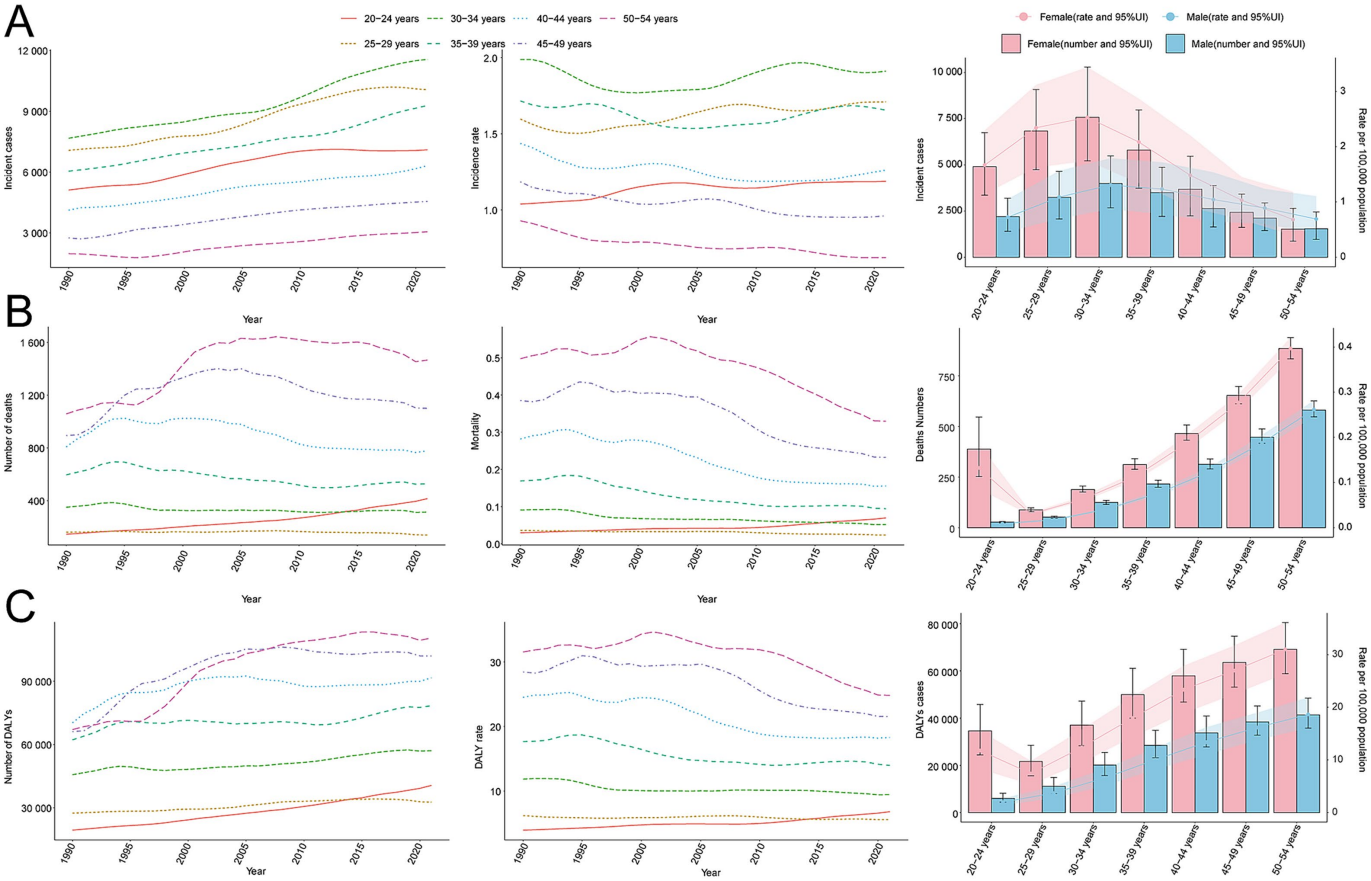
Trends in incidence, mortality, and disability-adjusted life years (DALYs) of multiple sclerosis by age and sex, 1990–2021. **(A)** Incidence cases and rate. **(B)** Mortality cases and rate. **(C)** DALYs cases and rate.

**Figure 4:**
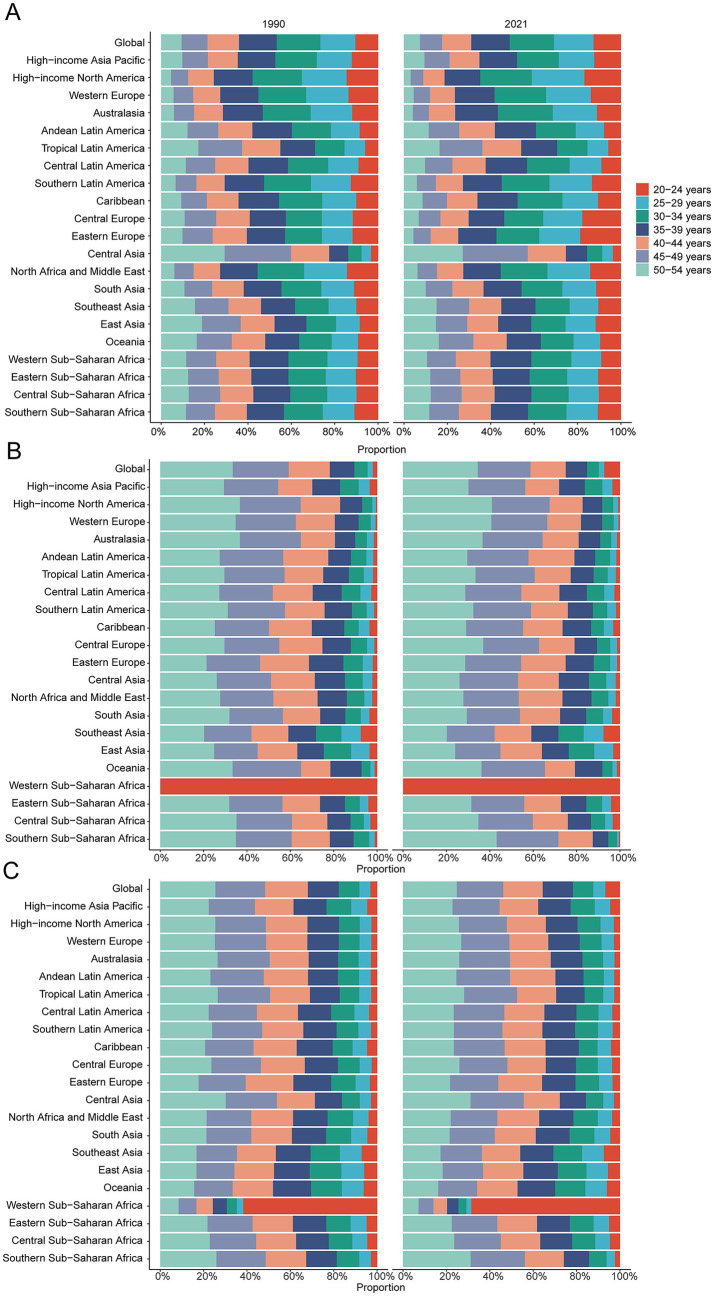
Age-specific percentages of multiple sclerosis incidence, mortality, and disability-adjusted life years (DALYs) in 1990 and 2021. **(A)** Incidence. **(B)** Deaths. **(C)** DALYs.

Comprehensive data analysis demonstrates significant dynamic shifts in the global epidemiology of MS, with incidence rates declining initially and subsequently rising. The lowest APC globally was observed from 1992 to 1995 at −0.72% (95% CI, −0.83% to −0.61%). For males, the lowest APC occurred between 1990 and 1993 (−1.01%; 95% CI, −1.15% to −0.87%), while females exhibited their lowest APC from 1990 to 1994 (−1.09%; 95% CI, −1.14% to −1.03%) (Figure 1A). Additionally, a global incidence nadir was observed in 2007 at 0.16 per 100,000 population (95% UI, 0.15–0.17). Specifically, incidence for males reached a low point in 2010 at 0.11 per 100,000 (95% UI, 0.10–0.11), while females reached their lowest incidence in 2007 at 0.20 per 100,000 (95% UI, 0.19–0.21) (Figure 1A). The total global cases of MS increased from 34,722.88 (95% UI, 29,443.06–40,689.55) in 1990 to 51,904.12 (95% UI, 44,998.80–59,073.37) in 2021, a 49.48% increase (95% UI, 44.34–55.15%). Nevertheless, the incidence rate decreased slightly from 1.44 per 100,000 (95% UI, 1.22–1.69) in 1990 to 1.38 per 100,000 (95% UI, 1.19–1.57) in 2021, with an EAPC of −0.08 (95% CI, −0.13 to −0.03) (Table 1 and Figures 2A–D). Incidence rates and cases were highest in the 30–34 age group and lowest among those aged 50–54 years from 1990 to 2021. Gender differences were evident, with females surpassing males in incidence rates from 20 to 49 years and converging in the 50–54 age bracket (Figures 3A, 4A).

**Table 1:** Incidence of multiple sclerosis between 1990 and 2021 at the global and regional level.

Location	1990		2021		1990–2021	
	Incident cases	Incidence rate	Incident cases	Incidence rate	Cases change	EAPC^a^
Global	34722.88(29443.06,40689.55)	1.44(1.22,1.69)	51904.12(44998.80,59073.37)	1.38(1.19,1.57)	49.48(44.34,55.15)	−0.08(−0.13,-0.03)
SDI						
High SDI	18364.77(15715.25,20969.49)	4.16(3.56,4.75)	22610.75(20267.46,24946.81)	4.38(3.93,4.83)	23.12(16.39,30.52)	0.24(0.18,0.30)
High-middle SDI	7381.88(6322.02,8527.68)	1.42(1.21,1.64)	8361.55(7261.65,9454.84)	1.28(1.11,1.45)	13.27(8.33,18.10)	−0.34(−0.37,-0.32)
Middle SDI	4706.87(3807.24,5812.71)	0.60(0.49,0.74)	9949.73(8248.56,11798.63)	0.81(0.67,0.96)	111.39(95.56,128.57)	1.15(1.09,1.20)
Low-middle SDI	3134.40(2483.58,3905.46)	0.67(0.53,0.83)	7866.61(6339.52,9524.56)	0.86(0.69,1.04)	150.98(136.16,168.12)	0.89(0.83,0.94)
Low SDI	1088.69(853.66,1370.76)	0.59(0.46,0.74)	3065.44(2451.16,3786.21)	0.68(0.54,0.84)	181.57(168.11,195.97)	0.45(0.40,0.49)
Regions						
Andean Latin America	78.59(60.37,99.23)	0.50(0.38,0.63)	231.46(182.53,282.05)	0.71(0.56,0.87)	194.53(174.50,220.95)	1.25(1.19,1.30)
Australasia	303.36(263.63,344.72)	3.01(2.62,3.43)	606.24(527.24,692.44)	4.16(3.61,4.75)	99.84(83.39,117.29)	1.11(0.86,1.35)
Caribbean	125.28(99.66,154.74)	0.79(0.63,0.97)	209.34(170.72,250.54)	0.91(0.74,1.09)	67.10(55.30,79.09)	0.40(0.34,0.47)
Central Asia	707.96(613.37,809.69)	2.38(2.06,2.72)	1167.46(1031.92,1320.62)	2.50(2.21,2.83)	64.91(55.95,74.35)	0.28(0.18,0.38)
Central Europe	1974.38(1726.03,2239.70)	3.33(2.91,3.78)	1527.61(1346.30,1712.20)	2.79(2.46,3.13)	−22.63(−25.30,-19.18)	−0.59(−0.62,-0.55)
Central Latin America	383.27(297.44,481.39)	0.56(0.44,0.71)	1059.75(862.27,1270.62)	0.85(0.69,1.02)	176.51(152.71,206.78)	1.40(1.28,1.51)
Central Sub-Saharan Africa	74.93(56.52,96.34)	0.37(0.28,0.48)	209.38(162.71,263.50)	0.39(0.30,0.48)	179.44(167.51,194.21)	0.09(0.03,0.15)
East Asia	1313.71(972.83,1744.96)	0.22(0.16,0.29)	1564.54(1177.01,1985.58)	0.21(0.16,0.27)	19.09(8.03,32.26)	−0.24(−0.37,-0.10)
Eastern Europe	2487.78(2074.63,2912.32)	2.26(1.88,2.64)	1781.54(1548.70,2001.35)	1.81(1.57,2.03)	−28.39(−33.50,-22.65)	−0.77(−0.81,-0.73)
Eastern Sub-Saharan Africa	280.88(214.30,363.10)	0.41(0.32,0.54)	705.87(549.87,896.30)	0.41(0.32,0.52)	151.31(143.11,160.56)	−0.07(−0.15,0.02)
High-income Asia Pacific	528.84(409.93,666.84)	0.60(0.47,0.76)	500.63(384.68,612.96)	0.59(0.46,0.73)	−5.33(−12.02,1.03)	−0.01(−0.06,0.03)
High-income North America	9335.54(7953.53,10708.91)	6.59(5.61,7.55)	10863.65(9928.20,11769.37)	6.46(5.91,7.00)	16.37(7.23,27.68)	0.21(0.08,0.34)
North Africa and Middle East	3313.35(2798.45,3865.61)	2.47(2.09,2.88)	9084.77(7655.09,10525.20)	2.93(2.47,3.39)	174.19(157.72,191.02)	0.75(0.68,0.81)
Oceania	5.64(4.08,7.66)	0.21(0.15,0.28)	12.63(9.37,16.67)	0.20(0.15,0.26)	123.99(112.85,133.90)	−0.17(−0.21,-0.12)
South Asia	2801.57(2191.06,3508.54)	0.61(0.48,0.77)	6402.58(5081.01,7886.84)	0.70(0.56,0.86)	128.54(116.38,141.69)	0.46(0.42,0.50)
Southeast Asia	494.19(363.67,660.35)	0.24(0.18,0.32)	858.67(651.14,1105.57)	0.24(0.18,0.31)	73.75(62.35,86.51)	−0.05(−0.09,-0.00)
Southern Latin America	374.96(306.63,452.39)	1.69(1.38,2.03)	546.47(451.96,653.97)	1.63(1.35,1.95)	45.74(38.25,53.33)	−0.13(−0.16,-0.09)
Southern Sub-Saharan Africa	120.57(93.25,153.96)	0.56(0.43,0.72)	226.79(176.49,284.53)	0.58(0.45,0.72)	88.10(77.37,100.10)	0.05(−0.03,0.14)
Tropical Latin America	921.37(748.28,1110.01)	1.35(1.10,1.63)	1963.42(1622.78,2313.49)	1.68(1.39,1.98)	113.10(96.26,133.67)	0.84(0.73,0.96)
Western Europe	8662.38(7442.16,9862.86)	4.58(3.94,5.22)	10964.14(9572.30,12318.88)	5.58(4.87,6.27)	26.57(20.01,33.44)	0.57(0.54,0.60)
Western Sub-Saharan Africa	434.35(341.19,542.90)	0.61(0.48,0.76)	1417.18(1156.14,1715.94)	0.75(0.61,0.91)	226.28(211.83,246.89)	0.72(0.69,0.76)

EAPC, estimated annual percentage change; SDI, Sociodemographic Index; UI, uncertainty interval. ^a^EAPC is expressed as 95% confidence interval.

#### Mortality

Contrasting incidence trends, global MS-related mortality rates initially rose and subsequently declined over the past three decades. The highest APC (1.98%; 95% CI, 0.92–3.05%) was observed between 1990 and 1993, peaking in 1993 at 0.18 deaths per 100,000 population (95% UI, 0.17–0.18). Peak mortality occurred for males in 1993 (0.14 per 100,000; 95% UI, 0.13–0.14) and for females in 2003 (0.22 per 100,000; 95% UI, 0.21–0.23) (Figure 1B). Global MS deaths rose from 4,008.58 (95% UI, 3,831.15–4,191.07) in 1990 to 4,738.38 (95% UI, 4,492.03–5,024.11) in 2021, an overall increase of 18.21% (95% UI, 11.60–25.45%). However, mortality rates decreased from 0.17 per 100,000 (95% UI, 0.16–0.17) in 1990 to 0.13 per 100,000 (95% UI, 0.12–0.13) in 2021 (EAPC, −1.21; 95% CI, −1.41 to −1.01) (Supplementary Table S1). Mortality varied by age group; it was highest among adults aged 50–54 years throughout 1990–2021, with female mortality consistently surpassing male mortality across all age groups (Figures 3B, 4B).

#### DALYs

Aligned with mortality trends, MS-related DALYs showed an initial increase followed by a decline over the past 30 years. The highest APC (1.05%; 95% CI, 0.52–1.59%) occurred from 1990 to 1993. DALY rates peaked globally in 2003 at 15.55 per 100,000 population (95% UI, 13.42–18.04), with female and male DALY rates peaking in 2003 (20.18 per 100,000; 95% UI, 17.39–23.42) and 1993 (11.25 per 100,000; 95% UI, 9.75–13.01), respectively (Figure 1C). Global DALYs increased by 43.22%, from 358,185.00 (95% UI, 307,223.63–418,638.58) in 1990 to 512,985.58 (95% UI, 428,133.20–610,308.62) in 2021. Despite this increase, DALY rates declined from 14.90 per 100,000 (95% UI, 12.78–17.42) to 13.61 per 100,000 (95% UI, 11.36–16.19) (EAPC, −0.45; 95% CI, −0.55 to −0.35) (Supplementary Table S2). DALYs were highest among adults aged 50–54 in 2021 and lowest in the 25–29 age group. Female DALY rates consistently exceeded male rates across all age groups (Figures 3C, 4C).

### SDI regional trends

#### Incidence

**Figure 5:**
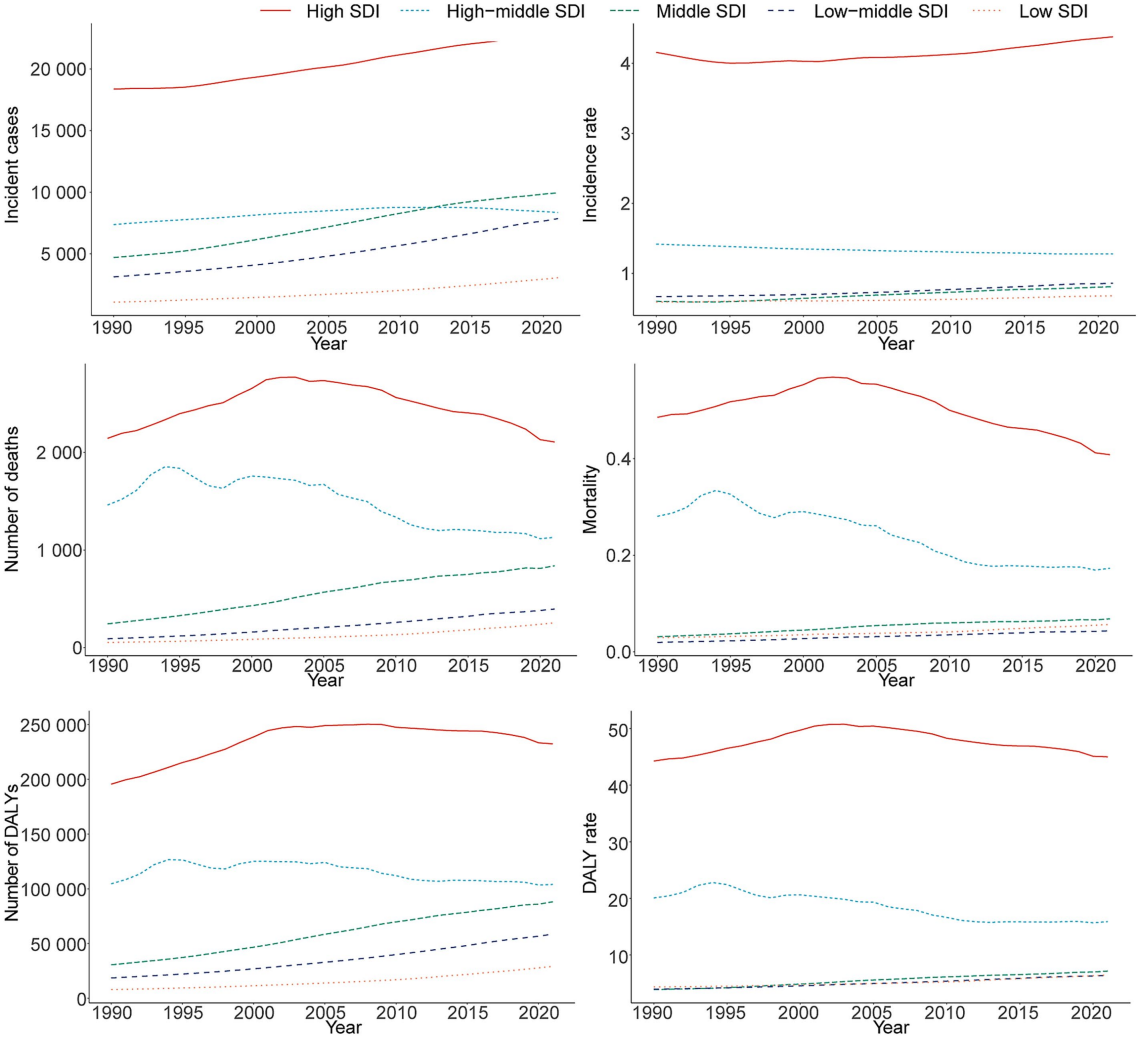
Epidemiologic trends in multiple sclerosis incidence, mortality, and disability-adjusted life years (DALYs) rates across five sociodemographic index (SDI) areas from 1990 to 2021.

In 2021, the High SDI region reported the highest incidence of MS with 22,610.75 cases (95% UI, 20,267.46–24,946.81), marking a 23.12% (95% UI, 16.39–30.52%) increase from 1990. Correspondingly, the incidence rate reached 4.38 per 100,000 individuals (95% UI, 3.93–4.83), with an EAPC of 0.24 (95% CI, 0.18–0.30) (Table 1 and Figure 5).

#### Mortality

Consistent with incidence trends, the High SDI region exhibited the highest MS-related mortality in 2021, with 2,107.28 deaths (95% UI, 2,029.19–2,176.77) and a mortality rate of 0.41 per 100,000 (95% UI, 0.39–0.42). The Low SDI region experienced the largest increase in deaths, totaling 370.57 cases (95% UI, 213.04–690.81) (Supplementary Table S1 and Figure 5).

#### DALYs

The High SDI region also recorded the highest MS-related DALYs at 232,327.05 (95% UI, 192,195.98–274,268.61), corresponding to a DALY rate of 45.00 per 100,000 (95% UI, 37.22–53.12). The greatest increase in DALYs from 1990 to 2021 occurred in the Low-middle SDI region, rising by 263.44% (95% UI, 196.39–347.66) (Supplementary Table S2 and Figure 5).

### Geographic regional trends

#### Incidence

**Figure 6:**
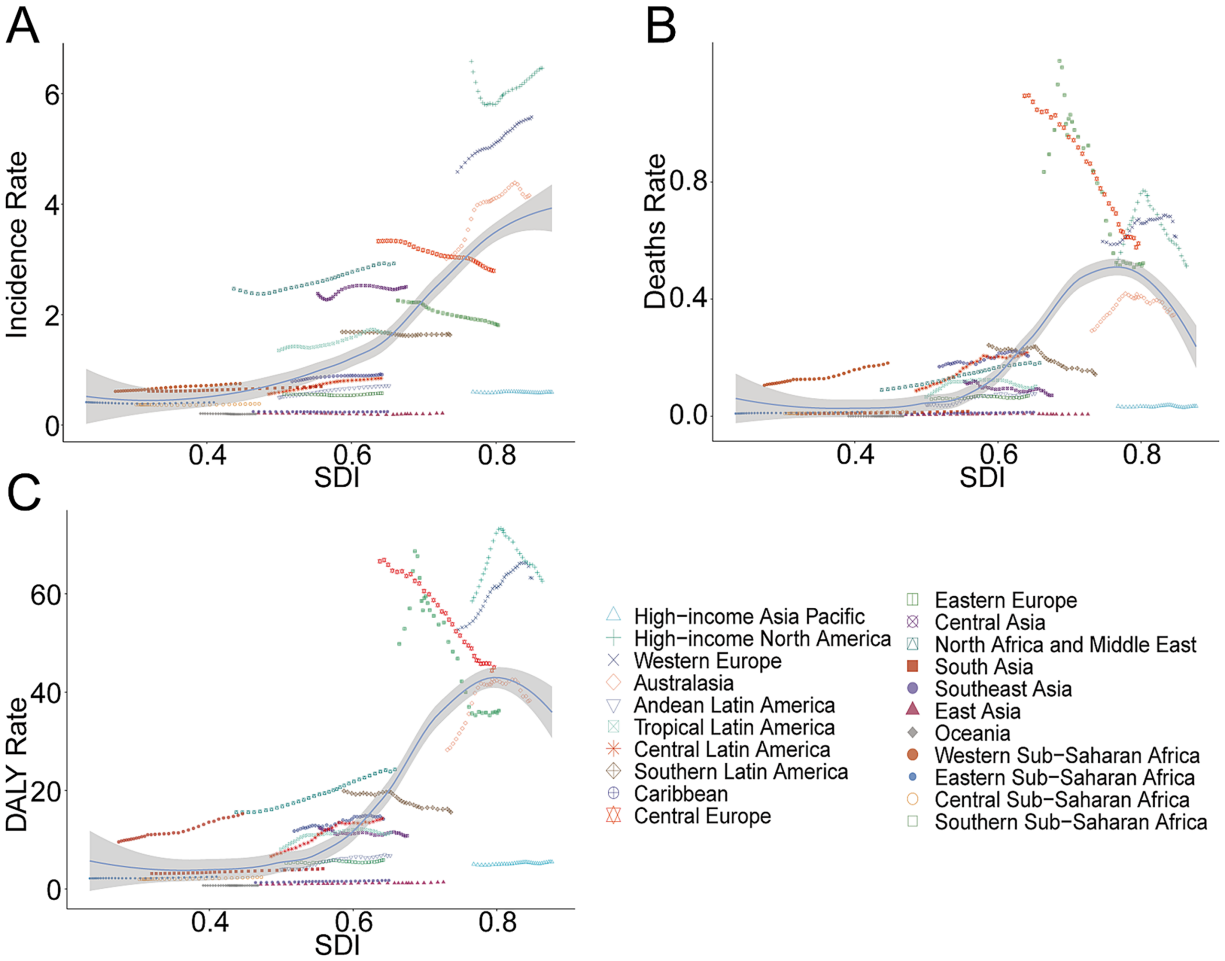
Association between incidence, mortality, and disability-adjusted life year (DALY) rates of multiple sclerosis and regional sociodemographic index, 1990–2021. **(A)** Incidence rate. **(B)** Mortality. **(C)** DALY rate.

Among the 21 geographic regions in 2021, Oceania reported the lowest MS incidence with 12.63 cases (95% UI, 9.37–16.67), while Western Europe had the highest at 10,964.14 cases (95% UI, 9,572.30–12,318.88). High-income North America had the highest incidence rate, whereas Oceania had the lowest. With a global SDI of 0.67, nine regions (e.g., High-income North America, Western Europe) surpassed the global mean incidence rate (1.38), while 12 regions (e.g., High-income Asia Pacific, South Asia) were below it (Table 1 and Figure 6A).

#### Mortality

Western Europe had the highest MS-related mortality in 2021, with 1,203.91 deaths (95% UI, 1,139.35–1,264.34), and the highest mortality rate (0.61 per 100,000; 95% UI, 0.58–0.64). Compared to the global average mortality rate of 0.13, 10 regions exceeded this benchmark, whereas 11 remained below it (Supplementary Table S1 and Figure 6B).

#### DALYs

Western Europe also led in MS-related DALYs with 124,183.10 (95% UI, 102,884.54–145,188.06) and recorded the highest DALY rate of 63.18 per 100,000 (95% UI, 52.35–73.87). Ten regions had DALY rates higher than the global mean (13.61), while 11 regions remained below (Supplementary Table S2 and Figure 6C).

### National trends

#### Incidence

Among 204 countries in 2021, the United States reported the most MS incident cases at 9,388.21 (95% UI, 8,469.23–10,275.59), representing a 14.84% (95% UI, 4.89–27.67%) increase since 1990. Sweden had the highest national incidence rate at 10.12 per 100,000 (95% UI, 8.69–11.66), with an EAPC of 0.24 (95% CI, 0.10–0.38) (Figures 2A–D). Globally, 83 countries exceeded the average incidence rate of 1.38, while 121 countries remained below.

#### Mortality

The United States also had the highest number of MS-related deaths in 2021 at 757.44 (95% UI, 722.91–791.99). The United Kingdom exhibited the highest mortality rate of 1.09 per 100,000 (95% UI, 1.05–1.13), significantly above the global average rate of 0.13, surpassed by 97 countries (Supplementary Tables S1, S3).

#### DALYs

The United States recorded the highest number of MS-related DALYs at 91,780.46 (95% UI, 75,626.43–109,300.06). The United Kingdom reported the highest national DALY rate at 95.49 per 100,000 (95% UI, 80.83–110.18). Worldwide, 99 countries exceeded the global average DALY rate of 13.61, while 105 countries were below this level (Supplementary Tables S2, S4).

#### BAPC

**Figure 7:**
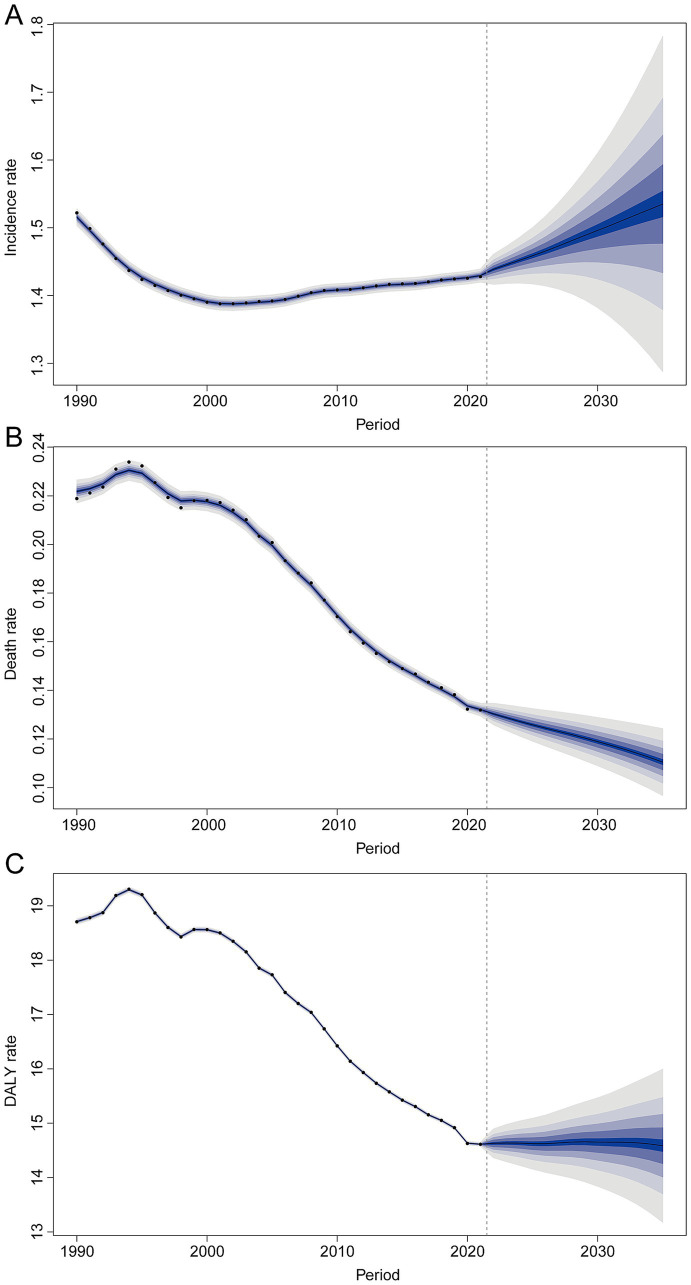
Projections of future trends in multiple sclerosis globally up to 2035. **(A)** Incidence rate. **(B)** Mortality. **(C)** DALY rate.

When projecting the burden of MS from 2021 to 2035 using the BAPC model, we estimated that by 2035 the incidence rate would be 1.54 per 100,000 (95% CI, 1.24–1.83), mortality rate 0.11 per 100,000 (95% CI, 0.09–0.13), and DALY rate 14.59 per 100,000 (95% CI, 12.91–16.26) (Figures 7A–C). These forecasts indicate a declining trend in the global burden of MS.

## Discussion

Over the past three decades, MS prevalence has increased globally, progressively emerging as a significant public health concern due to escalating medical and societal costs. This study analyzed incidence, mortality, and DALYs related to MS among adults aged 20–54 across all GBD regions and countries from 1990 to 2021, employing BAPC models to project MS disease burden up to 2035. Our findings reinforce earlier reports, revealing rising MS burdens among adults in specific regions and countries.

In 2021, an estimated 51,904 individuals developed MS, and 4,738 died from it globally. Notably, however, the rates of MS incidence and mortality have remained relatively stable or even slightly declined over this period. This suggests that population growth and aging, alongside improved case ascertainment, largely explain the increase in raw case numbers, rather than a universal surge in per-capita disease risk. Consistent with this, a prior analysis found a ~10% rise in global age-standardized MS prevalence from 1990 to 2016, accompanied by an 11.5% decrease in the MS mortality rate (8). In other words, the incidence of MS is higher today than in 1990, but the mortality rate (after adjusting for demographic structure) is lower, which may be closely associated with advancements in medical technology (24).

One of the most positive trends in MS epidemiology is the declining mortality and slowing disability progression, attributable to advances in medical care (25). The introduction of disease-modifying therapies (DMTs) since the mid-1990s (e.g., beta interferons, glatiramer acetate, and, more recently, monoclonal antibodies) and improvements in symptomatic management have significantly altered the disease course (26). Patients today experience fewer relapses and extended survival compared to past decades. Long-term population studies demonstrate a clear rise in MS survival over time (27). Likewise, a study in Canada found that MS patients treated with interferon-beta had a ~30% lower mortality risk than untreated patients over a long follow-up (28). They mean that MS is increasingly managed as a chronic lifelong condition rather than a rapidly fatal one. It highlights a shifting public health challenge: providing long-term support and rehabilitation for the growing cohort of MS survivors, many of whom have decades of living with disability ahead.

Another major driver of changing MS trends is the vast improvement in diagnostic capacity and criteria. The late 20th and early 21st centuries saw the widespread adoption of MRI technology and the successive revisions of MS diagnostic criteria that allow earlier and more sensitive detection of the disease. This has led to higher reported incidence and prevalence simply by identifying cases that would have been missed or delayed in earlier eras. A striking example comes from Finland: the national MS incidence nearly tripled – from about 3.7 per 100,000 in the 1980s (pre-MRI era) to 9.2 per 100,000 in the 2000s – after MRI-based criteria were incorporated (29). This pattern indicates that much of the “increase” in MS incidence in developed countries during the 1990s–2000s was driven by improved case ascertainment. Greater awareness among physicians, the proliferation of neurologists and MRI scanners, and standardized diagnostic guidelines all contributed to more complete and earlier recognition of MS. In lower-resource settings, diagnostic improvements are also key to explaining rising case numbers – many countries that once lacked MRI or trained neurologists are now detecting MS cases that went unrecognized in the past. Thus, part of the observed global rise in MS (especially in places like India or Latin America) may reflect closing the diagnostic gap. While better diagnostics inflate incidence in the short term, they are beneficial as they enable the timely initiation of therapy, which can improve long-term outcomes. As diagnostic practices stabilize worldwide, we may observe a true underlying incidence that is steadier, driven mostly by genuine changes in risk exposure rather than ascertainment.

Changes in the environment and lifestyle over the last few decades are likely contributing to the evolving patterns of MS worldwide. MS is known to arise from a complex interaction between genetic susceptibility and environmental exposures (30). Established risk factors include Epstein–Barr virus (EBV) infection, low sunlight/vitamin D levels, cigarette smoking, and obesity (particularly in early life) (31–34). Many of these factors have been in flux globally since 1990. For instance, urbanization and indoor lifestyles have reduced sun exposure for many populations, potentially increasing vitamin D deficiency, a condition strongly linked to higher MS risk (35). Dietary changes and more sedentary living have driven up childhood and adolescent obesity rates worldwide, and evidence suggests that childhood obesity may predispose individuals to MS in later life (36). Cigarette smoking, another known MS risk factor (37), has seen divergent trends: it has declined in some high-income countries (possibly mitigating MS risk slightly there), but in many low- and middle-income countries, smoking rates have risen or shifted to include more women. Such trends could help explain the rising MS incidence among females in those regions. The combination of modern lifestyles and persistent infections creates a multifaceted risk landscape for MS. It is conceivable that the increasing MS incidence observed in many regions results from a convergence of these factors: more people today have grown up with risk-enhancing exposures (such as high BMI and smoking) while also experiencing delayed EBV infection and vitamin D insufficiency. This aligns with the notion of an “epidemiological transition” for MS in low-prevalence regions – as countries industrialize, they begin to acquire the environmental risk profile that facilitates higher MS rates.

The gender disparity in MS provides another important clue to shifting risk factors. MS has a well-established female predominance, and this gap has widened in recent decades. Globally, women now outnumber men roughly 3 to 1 among MS patients (38). Furthermore, epidemiological analyses show that the increasing incidence of MS over the late 20th century was largely driven by rising cases in women, particularly for the relapsing–remitting form of MS (39). Many Western countries have documented a secular increase in the female-to-male ratio of MS. For example, Sweden saw a significant uptick in the proportion of female MS cases throughout the 20th century, mirroring trends observed in North America and Europe (40). The reasons for this female-specific rise are not fully settled, but changes in environmental exposures that differentially affect women are strongly suspected (41).

The evolving epidemiology of MS carries several implications for global health policy and neurology services. First, MS should be recognized as a significant contributor to the burden of neurological disease worldwide, even in regions where it was once considered rare. In 2016, MS ranked among the top 10 causes of neurologic disability globally (42), and its prominence will likely grow as other neurological conditions (e.g., stroke) are addressed and populations age. Health policymakers in high-burden countries – such as those in Northern Europe, North America, and Australasia – face the challenge of providing lifelong care (including expensive DMTs and rehabilitation) to a growing MS patient population. The substantial MS burden in these regions calls for sustained investment in neurology infrastructure and innovative care models.

In summary, the period from 1990 to 2021 has witnessed important shifts in the global landscape of multiple sclerosis. The overall picture is one of a growing burden, but also meaningful progress: patients are being diagnosed earlier, living longer, and – in many cases – achieving better control of disease activity than in the past. Meanwhile, the disease’s reach has extended into regions previously thought to be largely unaffected, underscoring that MS is a global health concern and not merely a Western problem.

### Limitations

This study has several limitations. First, as a cross-sectional analysis based on the GBD database, the validity and accuracy of the data are inevitably influenced by variations in reporting standards, diagnostic definitions, and methodologies across different national statistical and health agencies. Such inconsistencies may result in the underestimation of the true incidence of MS. Second, data availability in under-resourced regions is a significant concern, as a substantial proportion of undiagnosed MS cases may not be adequately captured, potentially distorting the representation of the actual disease burden.

## Conclusion

Our findings highlight a complex global landscape of MS, characterized by fluctuating incidence and improving mortality and DALY trends, albeit with notable regional disparities. Targeted strategies tailored to high-risk demographics and regions remain crucial to further alleviate the global burden of MS.

## Data Availability

The datasets presented in this study can be found in online repositories. The names of the repository/repositories and accession number(s) can be found: https://vizhub.healthdata.org/gbd-results/.

## Footnotes

^1^ https://vizhub.healthdata.org/gbd-results/
