# Supplementary material for: Emerging epidemiological trends of multiple sclerosis among adults aged 20–54 years, 1990–2021, with projections to 2035: a systematic analysis for the global burden of disease study 2021
Source: Front Neurol. 2025 Jul 10;16:1616245. doi: 10.3389/fneur.2025.1616245 (PMC12286822; doi:10.3389/fneur.2025.1616245)
Supplement: Supplementary file 1 [file Table_1.DOCX]

Table S1. Mortality of multiple sclerosis between 1990 and 2021 at the global and regional level.

|  | 1990 |  |  | 2021 |  |  | 1990-2021 |  |
| --- | --- | --- | --- | --- | --- | --- | --- | --- |
| **Location** | Death Cases | Death Rate |  | Death Cases | Death Rate |  | Cases change | EAPC |
| Global | 4008.58(3831.15,4191.07) | 0.17(0.16,0.17) |  | 4738.38(4492.03,5024.11) | 0.13(0.12,0.13) |  | 18.21(11.60,25.45) | -1.21(-1.41,-1.01) |
| **SDI** |  |  |  |  |  |  |  |  |
| High SDI | 2144.76(2102.02,2190.81) | 0.49(0.48,0.50) |  | 2107.28(2029.19,2176.77) | 0.41(0.39,0.42) |  | -1.75(-6.10,2.28) | -0.62(-0.90,-0.33) |
| High-middle SDI | 1461.43(1400.42,1541.59) | 0.28(0.27,0.30) |  | 1131.00(1037.89,1226.66) | 0.17(0.16,0.19) |  | -22.61(-30.25,-14.58) | -2.36(-2.66,-2.07) |
| Middle SDI | 244.53(199.24,286.79) | 0.03(0.03,0.04) |  | 839.65(771.64,923.45) | 0.07(0.06,0.08) |  | 243.37(180.74,329.56) | 2.57(2.33,2.81) |
| Low-middle SDI | 91.21(57.00,124.09) | 0.02(0.01,0.03) |  | 396.81(307.55,500.48) | 0.04(0.03,0.05) |  | 335.05(217.13,578.54) | 2.66(2.53,2.79) |
| Low SDI | 54.33(20.39,88.30) | 0.03(0.01,0.05) |  | 255.67(126.43,380.65) | 0.06(0.03,0.08) |  | 370.57(213.04,690.81) | 2.10(2.01,2.20) |
| **Regions** |  |  |  |  |  |  |  |  |
| Andean Latin America | 6.22(4.71,8.05) | 0.04(0.03,0.05) |  | 25.17(18.08,34.91) | 0.08(0.06,0.11) |  | 304.52(191.31,456.68) | 2.97(2.51,3.44) |
| Australasia | 29.41(26.81,32.34) | 0.29(0.27,0.32) |  | 50.43(44.21,56.79) | 0.35(0.30,0.39) |  | 71.51(48.16,99.73) | 0.44(0.07,0.81) |
| Caribbean | 26.89(24.64,29.72) | 0.17(0.16,0.19) |  | 47.16(39.71,57.12) | 0.21(0.17,0.25) |  | 75.37(47.06,107.80) | 0.87(0.72,1.02) |
| Central Asia | 35.22(31.06,39.69) | 0.12(0.10,0.13) |  | 33.26(24.82,41.32) | 0.07(0.05,0.09) |  | -5.57(-30.70,20.36) | -1.42(-1.65,-1.19) |
| Central Europe | 648.68(617.30,688.64) | 1.09(1.04,1.16) |  | 322.55(288.17,358.85) | 0.59(0.53,0.66) |  | -50.28(-56.18,-43.95) | -2.29(-2.42,-2.16) |
| Central Latin America | 59.44(57.25,61.78) | 0.09(0.08,0.09) |  | 271.46(240.16,306.57) | 0.22(0.19,0.25) |  | 356.72(296.88,423.83) | 3.08(2.68,3.49) |
| Central Sub-Saharan Africa | 1.87(0.77,3.33) | 0.01(0.00,0.02) |  | 8.32(4.13,13.32) | 0.02(0.01,0.02) |  | 345.60(169.56,622.06) | 1.61(1.43,1.79) |
| East Asia | 27.25(16.48,40.36) | 0.00(0.00,0.01) |  | 48.74(37.73,61.67) | 0.01(0.01,0.01) |  | 78.89(6.01,192.65) | 1.02(0.46,1.58) |
| Eastern Europe | 920.34(887.60,959.28) | 0.83(0.80,0.87) |  | 514.95(452.42,585.68) | 0.52(0.46,0.59) |  | -44.05(-51.31,-35.48) | -2.94(-3.46,-2.41) |
| Eastern Sub-Saharan Africa | 6.04(1.85,10.21) | 0.01(0.00,0.02) |  | 25.08(10.06,38.38) | 0.01(0.01,0.02) |  | 315.15(183.32,547.36) | 1.39(1.29,1.50) |
| High-income Asia Pacific | 29.63(28.05,31.10) | 0.03(0.03,0.04) |  | 28.88(26.89,31.26) | 0.03(0.03,0.04) |  | -2.55(-10.43,6.80) | 0.11(-0.17,0.39) |
| High-income North America | 731.19(710.22,753.94) | 0.52(0.50,0.53) |  | 862.30(825.25,900.13) | 0.51(0.49,0.54) |  | 17.93(11.90,24.88) | -0.35(-0.85,0.15) |
| North Africa and Middle East | 121.07(68.36,176.72) | 0.09(0.05,0.13) |  | 564.10(468.91,663.81) | 0.18(0.15,0.21) |  | 365.93(217.51,694.20) | 2.47(2.32,2.62) |
| Oceania | 0.00(0.00,0.00) | 0.00(0.00,0.00) |  | 0.00(0.00,0.00) | 0.00(0.00,0.00) |  | 163.89(71.91,293.18) | 0.23(0.08,0.39) |
| South Asia | 36.96(15.70,61.24) | 0.01(0.00,0.01) |  | 146.54(94.14,193.55) | 0.02(0.01,0.02) |  | 296.43(165.24,621.01) | 2.25(2.18,2.33) |
| Southeast Asia | 14.38(9.97,18.63) | 0.01(0.00,0.01) |  | 48.08(40.16,55.79) | 0.01(0.01,0.02) |  | 234.28(149.68,379.16) | 1.94(1.73,2.14) |
| Southern Latin America | 53.89(49.90,57.52) | 0.24(0.22,0.26) |  | 47.81(42.49,53.33) | 0.14(0.13,0.16) |  | -11.29(-21.99,1.52) | -1.70(-1.93,-1.46) |
| Southern Sub-Saharan Africa | 12.60(9.72,15.61) | 0.06(0.05,0.07) |  | 27.57(21.11,34.65) | 0.07(0.05,0.09) |  | 118.83(61.98,180.39) | 0.34(0.16,0.53) |
| Tropical Latin America | 44.94(42.78,46.89) | 0.07(0.06,0.07) |  | 118.84(111.37,126.06) | 0.10(0.10,0.11) |  | 164.42(142.82,187.48) | 0.68(0.05,1.31) |
| Western Europe | 1127.44(1095.80,1163.46) | 0.60(0.58,0.62) |  | 1203.91(1139.35,1264.34) | 0.61(0.58,0.64) |  | 6.78(0.06,12.80) | 0.41(0.26,0.57) |
| Western Sub-Saharan Africa | 75.13(39.77,113.42) | 0.11(0.06,0.16) |  | 343.21(209.38,498.65) | 0.18(0.11,0.26) |  | 356.85(181.08,708.70) | 1.72(1.57,1.88) |
