# Supplementary material for: Emerging epidemiological trends of multiple sclerosis among adults aged 20–54 years, 1990–2021, with projections to 2035: a systematic analysis for the global burden of disease study 2021
Source: Front Neurol. 2025 Jul 10;16:1616245. doi: 10.3389/fneur.2025.1616245 (PMC12286822; doi:10.3389/fneur.2025.1616245)
Supplement: Supplementary file 2 [file Table_2.DOCX]

Table S2. DALYs of multiple sclerosis between 1990 and 2021 at the global and regional level.

|  | 1990 |  |  | 2021 |  |  | 1990-2021 |  |
| --- | --- | --- | --- | --- | --- | --- | --- | --- |
| Location | DALYs Cases | DALY Rate |  | DALYs Cases | DALY Rate |  | Cases change | EAPC |
| Global | 358185.00(307223.63,418638.58) | 14.90(12.78,17.42) |  | 512985.58(428133.20,610308.62) | 13.61(11.36,16.19) |  | 43.22(36.79,48.94) | -0.45(-0.55,-0.35) |
| **SDI** |  |  |  |  |  |  |  |  |
| High SDI | 195532.25(165935.48,227767.19) | 44.26(37.56,51.56) |  | 232327.05(192195.98,274268.61) | 45.00(37.22,53.12) |  | 18.82(13.46,23.97) | 0.01(-0.16,0.18) |
| High-middle SDI | 104570.98(93342.11,117810.78) | 20.08(17.92,22.62) |  | 103954.15(88193.35,121530.03) | 15.90(13.49,18.58) |  | -0.59(-8.39,6.38) | -1.28(-1.46,-1.10) |
| Middle SDI | 30646.61(24663.48,39014.32) | 3.91(3.14,4.97) |  | 88164.04(72595.31,107598.82) | 7.17(5.90,8.75) |  | 187.68(162.09,218.85) | 2.09(1.98,2.20) |
| Low-middle SDI | 18599.83(13962.13,24879.85) | 3.96(2.97,5.30) |  | 58659.28(46415.69,75189.26) | 6.41(5.07,8.21) |  | 215.38(177.08,264.49) | 1.63(1.61,1.66) |
| Low SDI | 8038.65(5224.45,11270.39) | 4.36(2.83,6.11) |  | 29215.83(19654.91,39816.29) | 6.48(4.36,8.83) |  | 263.44(196.39,347.66) | 1.29(1.20,1.38) |
| **Regions** |  |  |  |  |  |  |  |  |
| Andean Latin America | 621.05(494.89,794.26) | 3.96(3.16,5.06) |  | 2208.25(1713.85,2790.95) | 6.78(5.26,8.57) |  | 255.57(198.93,322.16) | 2.18(1.94,2.43) |
| Australasia | 2845.35(2357.54,3409.18) | 28.27(23.43,33.88) |  | 5585.30(4469.34,6817.58) | 38.29(30.64,46.74) |  | 96.30(70.00,123.34) | 0.97(0.60,1.35) |
| Caribbean | 1867.39(1675.54,2128.74) | 11.76(10.55,13.40) |  | 3261.50(2775.66,3899.23) | 14.22(12.10,17.00) |  | 74.66(54.51,97.33) | 0.77(0.66,0.88) |
| Central Asia | 3705.42(3058.03,4444.12) | 12.46(10.28,14.95) |  | 5043.54(3820.69,6479.56) | 10.82(8.19,13.89) |  | 36.11(16.87,52.90) | -0.31(-0.41,-0.21) |
| Central Europe | 39503.62(36343.53,43197.74) | 66.61(61.28,72.84) |  | 24633.29(21414.38,28170.63) | 45.05(39.16,51.52) |  | -37.64(-43.25,-32.10) | -1.48(-1.57,-1.40) |
| Central Latin America | 4512.19(3981.06,5160.31) | 6.62(5.84,7.57) |  | 17885.30(15601.69,20424.05) | 14.31(12.49,16.35) |  | 296.38(255.25,338.04) | 2.62(2.31,2.93) |
| Central Sub-Saharan Africa | 397.19(279.39,567.62) | 1.96(1.38,2.81) |  | 1298.95(935.71,1765.72) | 2.39(1.72,3.25) |  | 227.04(182.93,278.40) | 0.61(0.51,0.70) |
| East Asia | 5595.63(3914.13,7992.26) | 0.92(0.64,1.31) |  | 10153.04(7208.41,13586.09) | 1.38(0.98,1.85) |  | 81.45(55.71,105.79) | 1.08(0.97,1.20) |
| Eastern Europe | 54960.79(51143.87,59229.26) | 49.82(46.36,53.69) |  | 35688.06(30839.88,40208.10) | 36.22(31.30,40.81) |  | -35.07(-42.01,-28.02) | -2.20(-2.61,-1.79) |
| Eastern Sub-Saharan Africa | 1455.90(1009.06,2073.60) | 2.15(1.49,3.06) |  | 4281.87(2930.11,5881.75) | 2.50(1.71,3.43) |  | 194.10(167.97,235.20) | 0.40(0.30,0.50) |
| High-income Asia Pacific | 4429.41(3386.18,5764.87) | 5.03(3.84,6.54) |  | 4614.60(3465.12,6001.71) | 5.48(4.12,7.13) |  | 4.18(-0.13,8.40) | 0.35(0.26,0.44) |
| High-income North America | 82963.98(67997.25,99254.30) | 58.53(47.97,70.02) |  | 105169.13(86036.19,124958.26) | 62.57(51.19,74.34) |  | 26.76(18.89,35.53) | 0.12(-0.14,0.38) |
| North Africa and Middle East | 20938.04(16213.56,27511.65) | 15.61(12.09,20.51) |  | 75419.75(60916.27,92947.95) | 24.31(19.63,29.96) |  | 260.20(214.59,317.78) | 1.63(1.58,1.68) |
| Oceania | 18.94(11.47,28.77) | 0.70(0.42,1.06) |  | 45.18(27.84,68.20) | 0.72(0.44,1.08) |  | 138.60(130.71,148.58) | 0.03(-0.01,0.07) |
| South Asia | 14261.91(10049.29,19824.40) | 3.13(2.20,4.35) |  | 37542.23(27401.82,50039.23) | 4.10(3.00,5.47) |  | 163.23(143.71,183.95) | 0.92(0.87,0.97) |
| Southeast Asia | 2631.89(1916.82,3593.46) | 1.29(0.94,1.77) |  | 6157.78(4734.56,7850.05) | 1.74(1.34,2.21) |  | 133.97(110.39,165.67) | 0.87(0.80,0.93) |
| Southern Latin America | 4430.15(3775.75,5252.38) | 19.92(16.98,23.62) |  | 5222.20(4174.64,6377.83) | 15.60(12.47,19.06) |  | 17.88(4.52,31.03) | -0.81(-0.92,-0.70) |
| Southern Sub-Saharan Africa | 1133.49(911.64,1426.19) | 5.26(4.23,6.62) |  | 2319.86(1842.48,2897.07) | 5.90(4.69,7.37) |  | 104.66(73.73,134.09) | 0.18(0.06,0.30) |
| Tropical Latin America | 5507.19(4359.03,6939.69) | 8.08(6.40,10.18) |  | 13384.20(10821.29,16608.41) | 11.47(9.28,14.24) |  | 143.03(128.24,159.85) | 0.91(0.64,1.18) |
| Western Europe | 99602.04(85162.47,115393.64) | 52.71(45.07,61.07) |  | 124183.10(102884.54,145188.06) | 63.18(52.35,73.87) |  | 24.68(18.38,30.77) | 0.75(0.64,0.85) |
| Western Sub-Saharan Africa | 6803.44(4287.41,9482.11) | 9.55(6.02,13.31) |  | 28888.46(19613.96,39850.99) | 15.28(10.37,21.07) |  | 324.62(189.45,537.99) | 1.51(1.39,1.63) |
