# Supplementary material for: Emerging epidemiological trends of multiple sclerosis among adults aged 20–54 years, 1990–2021, with projections to 2035: a systematic analysis for the global burden of disease study 2021
Source: Front Neurol. 2025 Jul 10;16:1616245. doi: 10.3389/fneur.2025.1616245 (PMC12286822; doi:10.3389/fneur.2025.1616245)
Supplement: Supplementary file 3 [file Table_3.DOCX]

Table S3. Deaths of multiple sclerosis Between 1990 and 2021 at the Global and Regional Levels

| location | 1990 | |  | 2021 | |  | 1990-2021 | |
| --- | --- | --- | --- | --- | --- | --- | --- | --- |
|  | Death cases | Mortality |  | Death cases | Mortality |  | Cases change | EAPC |
| Afghanistan | 2.93(1.01,6.19) | 0.09(0.03,0.20) |  | 24.90(12.17,46.09) | 0.20(0.10,0.38) |  | 749.63(358.69,1713.82) | 3.22(2.92,3.52) |
| Albania | 18.11(12.38,25.00) | 1.20(0.82,1.65) |  | 13.22(7.30,23.09) | 1.05(0.58,1.83) |  | -27.02(-64.81,42.60) | -0.29(-0.54,-0.04) |
| Algeria | 6.26(3.28,10.68) | 0.06(0.03,0.11) |  | 49.76(31.39,76.25) | 0.23(0.14,0.35) |  | 695.12(302.60,1477.45) | 4.00(3.59,4.40) |
| American Samoa | 0.00(0.00,0.00) | 0.00(0.00,0.00) |  | 0.00(0.00,0.00) | 0.00(0.00,0.00) |  | 108.39(18.95,323.52) | 2.61(2.48,2.75) |
| Andorra | 0.16(0.09,0.28) | 0.52(0.29,0.89) |  | 0.30(0.15,0.51) | 0.66(0.34,1.15) |  | 83.60(-21.36,316.02) | 1.46(1.20,1.73) |
| Angola | 0.40(0.11,0.79) | 0.01(0.00,0.02) |  | 2.38(0.87,4.47) | 0.02(0.01,0.04) |  | 493.80(195.77,1317.10) | 2.20(2.06,2.34) |
| Antigua and Barbuda | 0.06(0.05,0.07) | 0.22(0.19,0.25) |  | 0.14(0.12,0.17) | 0.30(0.26,0.36) |  | 135.43(93.63,187.12) | 1.94(1.65,2.23) |
| Argentina | 40.18(36.59,43.81) | 0.28(0.25,0.30) |  | 35.63(31.29,40.30) | 0.16(0.14,0.18) |  | -11.33(-24.19,3.79) | -1.78(-2.03,-1.53) |
| Armenia | 1.11(0.95,1.28) | 0.07(0.06,0.08) |  | 0.84(0.70,1.01) | 0.06(0.05,0.07) |  | -24.66(-41.02,-3.19) | 1.38(0.37,2.40) |
| Australia | 23.44(21.00,25.99) | 0.28(0.25,0.31) |  | 41.06(35.80,47.04) | 0.34(0.29,0.39) |  | 75.21(49.15,110.21) | 0.59(0.25,0.92) |
| Austria | 22.62(20.46,25.01) | 0.57(0.52,0.63) |  | 25.09(22.02,28.39) | 0.59(0.52,0.66) |  | 10.93(-7.55,29.16) | 0.72(0.50,0.94) |
| Azerbaijan | 1.37(0.76,2.27) | 0.04(0.02,0.07) |  | 1.87(0.83,3.58) | 0.03(0.01,0.06) |  | 36.33(-44.59,219.33) | -0.14(-0.46,0.19) |
| Bahamas | 0.35(0.31,0.39) | 0.28(0.25,0.31) |  | 1.06(0.81,1.37) | 0.53(0.40,0.68) |  | 200.13(122.00,295.72) | 2.47(2.25,2.68) |
| Bahrain | 0.01(0.01,0.01) | 0.00(0.00,0.00) |  | 0.79(0.55,1.11) | 0.08(0.06,0.11) |  | 8317.99(5526.24,12439.26) | 9.57(7.60,11.58) |
| Bangladesh | 2.85(0.61,5.75) | 0.01(0.00,0.01) |  | 11.69(3.42,23.02) | 0.01(0.00,0.03) |  | 310.35(116.80,813.43) | 2.60(2.41,2.79) |
| Barbados | 0.39(0.35,0.42) | 0.32(0.29,0.35) |  | 0.71(0.53,0.92) | 0.50(0.37,0.65) |  | 84.87(34.38,145.77) | 2.05(1.79,2.31) |
| Belarus | 30.97(27.82,34.31) | 0.62(0.56,0.69) |  | 21.81(16.49,27.78) | 0.49(0.37,0.63) |  | -29.56(-47.48,-9.00) | -1.56(-2.15,-0.98) |
| Belgium | 27.36(25.06,29.53) | 0.56(0.51,0.60) |  | 35.52(31.81,39.59) | 0.69(0.62,0.77) |  | 29.80(14.13,47.48) | 0.59(0.29,0.88) |
| Belize | 0.03(0.03,0.04) | 0.05(0.04,0.05) |  | 0.24(0.20,0.29) | 0.11(0.09,0.14) |  | 643.12(502.57,812.71) | 3.23(2.83,3.63) |
| Benin | 1.68(0.44,4.24) | 0.10(0.03,0.26) |  | 8.75(2.60,22.14) | 0.17(0.05,0.43) |  | 420.27(45.60,2002.20) | 1.37(1.21,1.53) |
| Bermuda | 0.08(0.07,0.09) | 0.25(0.22,0.28) |  | 0.07(0.06,0.09) | 0.26(0.20,0.31) |  | -10.23(-30.07,13.77) | 0.19(-0.02,0.39) |
| Bhutan | 0.01(0.00,0.03) | 0.01(0.00,0.01) |  | 0.05(0.01,0.10) | 0.01(0.00,0.03) |  | 265.07(98.76,832.20) | 2.54(2.45,2.62) |
| Bolivia (Plurinational State of) | 1.30(0.55,2.48) | 0.05(0.02,0.10) |  | 5.40(2.40,10.43) | 0.09(0.04,0.18) |  | 316.36(104.02,755.31) | 1.89(1.85,1.93) |
| Bosnia and Herzegovina | 12.62(8.97,16.79) | 0.55(0.39,0.74) |  | 5.81(3.52,9.06) | 0.38(0.23,0.59) |  | -53.91(-74.23,-15.30) | -1.47(-1.73,-1.20) |
| Botswana | 0.03(0.01,0.06) | 0.01(0.00,0.01) |  | 0.12(0.07,0.20) | 0.01(0.01,0.02) |  | 260.79(70.54,816.06) | 1.20(0.95,1.46) |
| Brazil | 44.46(42.34,46.38) | 0.07(0.06,0.07) |  | 116.48(108.79,123.62) | 0.10(0.10,0.11) |  | 162.01(140.33,184.91) | 0.67(0.04,1.30) |
| Brunei Darussalam | 0.02(0.01,0.06) | 0.02(0.01,0.05) |  | 0.07(0.03,0.17) | 0.03(0.01,0.06) |  | 177.91(40.84,402.88) | 1.24(1.13,1.34) |
| Bulgaria | 36.60(32.66,40.53) | 0.90(0.81,1.00) |  | 26.68(21.26,32.39) | 0.86(0.68,1.04) |  | -27.09(-43.59,-8.14) | -0.42(-0.62,-0.22) |
| Burkina Faso | 3.07(0.51,8.21) | 0.10(0.02,0.26) |  | 13.62(3.18,39.46) | 0.16(0.04,0.47) |  | 343.82(9.30,1825.73) | 1.75(1.51,1.98) |
| Burundi | 0.16(0.05,0.31) | 0.01(0.00,0.02) |  | 0.50(0.12,0.90) | 0.01(0.00,0.02) |  | 213.57(59.74,447.19) | 0.14(-0.07,0.35) |
| Cabo Verde | 0.21(0.04,0.54) | 0.17(0.03,0.45) |  | 0.34(0.08,0.95) | 0.12(0.03,0.33) |  | 68.18(-54.15,578.37) | -0.62(-1.04,-0.19) |
| Cambodia | 0.12(0.05,0.23) | 0.00(0.00,0.01) |  | 0.61(0.25,1.11) | 0.01(0.00,0.01) |  | 403.10(169.63,841.42) | 2.70(2.41,2.99) |
| Cameroon | 5.04(1.83,11.22) | 0.13(0.05,0.30) |  | 25.73(7.79,66.35) | 0.20(0.06,0.52) |  | 410.83(12.10,1998.69) | 1.27(1.23,1.31) |
| Canada | 80.29(73.10,87.76) | 0.57(0.52,0.62) |  | 104.79(93.63,117.14) | 0.62(0.55,0.69) |  | 30.51(13.65,49.43) | 0.06(-0.41,0.52) |
| Central African Republic | 0.10(0.03,0.18) | 0.01(0.00,0.02) |  | 0.29(0.11,0.51) | 0.01(0.01,0.02) |  | 196.03(65.60,445.94) | 0.96(0.87,1.05) |
| Chad | 1.50(0.25,4.40) | 0.07(0.01,0.22) |  | 8.23(1.69,22.73) | 0.14(0.03,0.39) |  | 449.46(24.16,2203.92) | 2.22(2.13,2.31) |
| Chile | 8.08(7.37,8.88) | 0.13(0.12,0.14) |  | 7.22(6.41,8.01) | 0.08(0.07,0.08) |  | -10.64(-23.78,3.13) | -1.57(-1.80,-1.34) |
| China | 26.21(15.74,38.92) | 0.00(0.00,0.01) |  | 45.58(34.41,58.38) | 0.01(0.00,0.01) |  | 73.94(1.36,191.14) | 1.19(0.62,1.76) |
| Colombia | 11.94(10.97,13.03) | 0.08(0.08,0.09) |  | 33.05(26.50,40.45) | 0.13(0.11,0.16) |  | 176.81(116.63,237.48) | 1.69(1.19,2.19) |
| Comoros | 0.02(0.00,0.04) | 0.01(0.00,0.02) |  | 0.08(0.03,0.14) | 0.02(0.01,0.04) |  | 288.88(125.27,1080.06) | 1.53(1.22,1.83) |
| Congo | 0.11(0.07,0.18) | 0.01(0.01,0.02) |  | 0.58(0.32,1.01) | 0.02(0.01,0.04) |  | 409.57(165.23,846.42) | 1.92(1.76,2.07) |
| Cook Islands | 0.00(0.00,0.00) | 0.00(0.00,0.00) |  | 0.00(0.00,0.00) | 0.00(0.00,0.00) |  | 33.88(-36.52,210.82) | 1.46(1.25,1.68) |
| Costa Rica | 1.12(1.02,1.23) | 0.08(0.08,0.09) |  | 5.47(4.61,6.32) | 0.23(0.19,0.26) |  | 388.53(307.51,478.46) | 3.03(2.66,3.41) |
| Croatia | 4.91(1.31,12.85) | 0.11(0.03,0.28) |  | 19.19(5.53,53.01) | 0.16(0.05,0.45) |  | -54.41(-64.18,-42.88) | 1.78(1.64,1.92) |
| Cuba | 19.67(17.43,22.08) | 0.81(0.72,0.91) |  | 8.97(7.28,10.76) | 0.47(0.38,0.56) |  | 37.22(11.35,65.93) | -1.65(-1.80,-1.50) |
| Cyprus | 14.02(13.10,15.12) | 0.25(0.24,0.27) |  | 19.24(16.03,22.87) | 0.35(0.29,0.42) |  | 120.17(8.61,423.11) | 1.35(1.19,1.51) |
| Czechia | 1.04(0.45,1.95) | 0.28(0.12,0.51) |  | 2.30(1.40,3.72) | 0.32(0.19,0.51) |  | -53.24(-62.60,-42.22) | 0.45(0.28,0.62) |
| C么te d'Ivoire | 62.00(55.96,68.69) | 1.27(1.15,1.41) |  | 28.99(23.77,35.43) | 0.59(0.48,0.72) |  | 290.86(0.19,1426.44) | -2.69(-2.92,-2.47) |
| Democratic People's Republic of Korea | 0.61(0.23,1.17) | 0.01(0.00,0.01) |  | 1.32(0.58,2.44) | 0.01(0.00,0.02) |  | 116.23(11.00,369.70) | 1.49(1.38,1.60) |
| Democratic Republic of the Congo | 1.19(0.43,2.18) | 0.01(0.00,0.02) |  | 4.76(1.93,8.25) | 0.01(0.01,0.02) |  | 298.58(123.36,632.87) | 1.31(1.01,1.61) |
| Denmark | 30.85(27.17,35.06) | 1.19(1.05,1.35) |  | 23.93(20.81,26.94) | 0.91(0.79,1.02) |  | -22.44(-37.24,-7.52) | -0.80(-1.09,-0.50) |
| Djibouti | 0.02(0.00,0.03) | 0.01(0.00,0.02) |  | 0.12(0.04,0.24) | 0.02(0.01,0.04) |  | 619.46(302.55,1401.84) | 1.90(1.67,2.13) |
| Dominica | 0.02(0.01,0.02) | 0.06(0.04,0.07) |  | 0.04(0.02,0.06) | 0.12(0.07,0.19) |  | 124.11(31.56,263.73) | 2.93(2.64,3.21) |
| Dominican Republic | 1.56(1.13,1.99) | 0.05(0.04,0.07) |  | 4.52(2.83,6.66) | 0.08(0.05,0.12) |  | 190.19(64.33,429.68) | 2.24(1.96,2.51) |
| Ecuador | 2.20(2.04,2.38) | 0.05(0.05,0.06) |  | 7.93(5.99,10.09) | 0.09(0.07,0.12) |  | 259.70(171.31,374.78) | 3.16(2.27,4.05) |
| Egypt | 1.75(1.07,2.59) | 0.01(0.00,0.01) |  | 5.56(3.41,8.10) | 0.01(0.01,0.02) |  | 217.24(97.83,428.68) | 1.11(0.95,1.26) |
| El Salvador | 0.85(0.63,1.06) | 0.04(0.03,0.05) |  | 3.14(2.24,4.32) | 0.10(0.07,0.14) |  | 269.93(149.82,466.42) | 3.34(2.99,3.69) |
| Equatorial Guinea | 0.01(0.01,0.03) | 0.01(0.00,0.02) |  | 0.13(0.07,0.23) | 0.02(0.01,0.03) |  | 785.68(265.33,2656.93) | 2.38(2.18,2.57) |
| Eritrea | 0.15(0.04,0.29) | 0.01(0.00,0.02) |  | 0.63(0.20,1.31) | 0.02(0.01,0.04) |  | 331.44(113.65,860.40) | 1.93(1.75,2.11) |
| Estonia | 11.63(10.59,12.77) | 1.55(1.41,1.70) |  | 3.12(2.60,3.64) | 0.53(0.44,0.61) |  | -73.18(-78.29,-67.64) | -5.04(-5.56,-4.52) |
| Eswatini | 0.02(0.01,0.03) | 0.01(0.00,0.01) |  | 0.07(0.03,0.11) | 0.01(0.01,0.02) |  | 252.00(60.20,992.91) | 2.06(1.70,2.42) |
| Ethiopia | 1.73(0.53,3.27) | 0.01(0.00,0.02) |  | 5.77(1.62,9.81) | 0.01(0.00,0.02) |  | 233.91(92.70,423.86) | 0.53(0.32,0.74) |
| Fiji | 0.00(0.00,0.00) | 0.00(0.00,0.00) |  | 0.00(0.00,0.00) | 0.00(0.00,0.00) |  | 65.51(-16.18,272.14) | 0.73(0.57,0.88) |
| Finland | 17.79(16.27,19.32) | 0.70(0.64,0.76) |  | 14.99(13.24,16.71) | 0.63(0.56,0.70) |  | -15.75(-27.24,-2.79) | -0.33(-0.62,-0.04) |
| France | 127.64(117.90,139.81) | 0.46(0.43,0.50) |  | 135.02(117.17,154.25) | 0.47(0.41,0.54) |  | 5.79(-11.11,22.92) | 0.15(-0.24,0.54) |
| Gabon | 0.05(0.03,0.07) | 0.01(0.01,0.02) |  | 0.19(0.10,0.30) | 0.02(0.01,0.04) |  | 289.46(80.88,759.87) | 1.74(1.53,1.95) |
| Gambia | 0.39(0.08,1.09) | 0.11(0.02,0.30) |  | 2.40(0.68,5.92) | 0.25(0.07,0.61) |  | 516.18(28.22,2698.35) | 2.53(2.24,2.81) |
| Georgia | 1.17(0.96,1.43) | 0.04(0.04,0.05) |  | 0.72(0.57,0.91) | 0.04(0.03,0.06) |  | -38.32(-53.54,-17.78) | 2.30(0.87,3.75) |
| Germany | 308.82(285.85,332.86) | 0.74(0.69,0.80) |  | 235.16(210.71,263.13) | 0.62(0.56,0.69) |  | -23.85(-33.48,-13.53) | 0.05(-0.18,0.28) |
| Ghana | 10.49(3.44,24.49) | 0.18(0.06,0.43) |  | 35.41(11.03,87.84) | 0.23(0.07,0.58) |  | 237.66(-10.43,1234.52) | 0.81(0.71,0.90) |
| Greece | 15.26(13.77,16.86) | 0.31(0.28,0.34) |  | 29.54(26.17,33.00) | 0.64(0.57,0.72) |  | 93.65(67.83,126.68) | 2.68(2.59,2.77) |
| Greenland | 0.01(0.01,0.02) | 0.05(0.03,0.07) |  | 0.05(0.03,0.08) | 0.17(0.10,0.28) |  | 223.76(76.14,465.36) | 8.16(6.13,10.24) |
| Grenada | 0.09(0.08,0.11) | 0.28(0.25,0.32) |  | 0.19(0.15,0.23) | 0.36(0.29,0.43) |  | 100.76(59.42,155.65) | 1.48(1.12,1.84) |
| Guam | 0.00(0.00,0.00) | 0.00(0.00,0.00) |  | 0.00(0.00,0.00) | 0.00(0.00,0.00) |  | 20.76(-29.71,175.62) | 0.25(-0.42,0.93) |
| Guatemala | 1.93(1.78,2.09) | 0.07(0.06,0.07) |  | 7.88(6.55,9.38) | 0.11(0.09,0.13) |  | 308.72(231.33,397.26) | 1.57(1.13,2.01) |
| Guinea | 1.71(0.41,4.56) | 0.08(0.02,0.21) |  | 9.22(2.74,23.83) | 0.18(0.05,0.47) |  | 440.77(43.92,2167.78) | 2.83(2.71,2.95) |
| Guinea-Bissau | 0.52(0.16,1.28) | 0.15(0.05,0.36) |  | 1.87(0.63,4.65) | 0.22(0.08,0.56) |  | 259.69(-0.81,1174.67) | 1.40(1.21,1.60) |
| Guyana | 0.18(0.15,0.21) | 0.05(0.05,0.06) |  | 0.45(0.31,0.64) | 0.12(0.08,0.17) |  | 146.08(58.21,262.19) | 3.28(2.89,3.67) |
| Haiti | 2.51(1.10,4.73) | 0.10(0.04,0.19) |  | 8.80(3.90,17.06) | 0.14(0.06,0.28) |  | 251.01(76.33,589.55) | 1.27(1.18,1.37) |
| Honduras | 0.26(0.13,0.40) | 0.02(0.01,0.02) |  | 0.91(0.43,1.62) | 0.02(0.01,0.03) |  | 250.76(55.66,646.95) | 0.50(0.34,0.65) |
| Hungary | 54.27(48.66,60.42) | 1.10(0.99,1.23) |  | 28.51(23.75,33.77) | 0.63(0.52,0.74) |  | -47.46(-57.29,-34.36) | -2.29(-2.49,-2.09) |
| Iceland | 0.64(0.58,0.71) | 0.53(0.48,0.58) |  | 1.12(0.98,1.27) | 0.69(0.60,0.77) |  | 75.03(46.70,106.19) | 1.10(1.01,1.19) |
| India | 29.88(13.61,49.74) | 0.01(0.00,0.01) |  | 114.61(77.84,149.28) | 0.02(0.01,0.02) |  | 283.51(145.73,603.75) | 2.27(2.18,2.36) |
| Indonesia | 2.61(1.97,3.40) | 0.00(0.00,0.00) |  | 11.61(8.16,17.54) | 0.01(0.01,0.01) |  | 344.00(162.60,641.89) | 2.71(2.33,3.09) |
| Iran (Islamic Republic of) | 46.14(24.77,65.83) | 0.22(0.12,0.31) |  | 196.05(162.91,231.72) | 0.42(0.35,0.50) |  | 324.89(173.64,702.44) | 2.35(2.24,2.46) |
| Iraq | 3.46(1.35,6.32) | 0.05(0.02,0.09) |  | 17.73(10.96,27.61) | 0.09(0.06,0.14) |  | 412.18(135.18,1115.05) | 1.84(1.76,1.93) |
| Ireland | 11.34(10.27,12.54) | 0.71(0.64,0.79) |  | 17.31(14.51,19.86) | 0.75(0.63,0.86) |  | 52.69(23.74,83.60) | 0.47(0.34,0.59) |
| Israel | 3.42(3.14,3.74) | 0.16(0.15,0.17) |  | 7.87(7.02,8.83) | 0.19(0.17,0.21) |  | 130.34(100.23,170.22) | 0.72(0.45,0.99) |
| Italy | 110.37(105.22,115.57) | 0.39(0.38,0.41) |  | 122.43(112.32,131.32) | 0.46(0.42,0.49) |  | 10.93(1.74,20.70) | 0.94(0.69,1.19) |
| Jamaica | 0.60(0.54,0.66) | 0.06(0.06,0.07) |  | 2.23(1.59,3.03) | 0.15(0.11,0.21) |  | 272.07(170.27,413.22) | 3.23(2.74,3.71) |
| Japan | 21.41(20.79,22.07) | 0.03(0.03,0.04) |  | 21.53(20.47,22.47) | 0.04(0.04,0.04) |  | 0.57(-4.78,6.12) | 0.53(0.22,0.83) |
| Jordan | 2.89(1.95,4.22) | 0.20(0.14,0.30) |  | 13.74(9.55,18.90) | 0.22(0.16,0.31) |  | 376.24(171.62,722.56) | 0.23(-0.07,0.53) |
| Kazakhstan | 23.25(19.35,27.77) | 0.30(0.25,0.36) |  | 14.89(9.30,21.46) | 0.16(0.10,0.24) |  | -35.95(-61.26,-4.45) | -2.50(-2.87,-2.12) |
| Kenya | 0.60(0.24,0.90) | 0.01(0.00,0.01) |  | 3.46(2.38,4.85) | 0.02(0.01,0.02) |  | 479.75(271.93,1216.70) | 2.34(2.23,2.45) |
| Kiribati | 0.00(0.00,0.00) | 0.00(0.00,0.00) |  | 0.00(0.00,0.00) | 0.00(0.00,0.00) |  | 36.63(-39.14,247.53) | -1.60(-2.12,-1.08) |
| Kuwait | 0.00(0.00,0.00) | 0.00(0.00,0.00) |  | 1.86(1.52,2.25) | 0.06(0.05,0.07) |  | 355313.15(280698.08,433832.22) | 21.23(15.09,27.71) |
| Kyrgyzstan | 1.20(1.04,1.38) | 0.07(0.06,0.07) |  | 1.56(1.22,1.97) | 0.05(0.04,0.06) |  | 29.54(-1.70,68.75) | 0.10(-1.08,1.28) |
| Lao People's Democratic Republic | 0.05(0.02,0.11) | 0.00(0.00,0.01) |  | 0.27(0.11,0.49) | 0.01(0.00,0.01) |  | 442.91(176.71,1089.44) | 2.61(2.30,2.92) |
| Latvia | 22.87(20.86,25.20) | 1.79(1.63,1.97) |  | 7.44(6.19,8.71) | 0.90(0.75,1.06) |  | -67.45(-73.71,-59.91) | -3.61(-4.07,-3.15) |
| Lebanon | 1.25(0.69,2.15) | 0.10(0.05,0.17) |  | 3.55(2.35,5.05) | 0.12(0.08,0.17) |  | 184.89(44.63,412.50) | 0.77(0.65,0.89) |
| Lesotho | 0.03(0.01,0.06) | 0.01(0.00,0.01) |  | 0.09(0.05,0.16) | 0.01(0.01,0.02) |  | 192.97(25.84,1057.59) | 2.36(1.91,2.82) |
| Liberia | 0.89(0.26,2.29) | 0.10(0.03,0.25) |  | 4.36(1.26,10.81) | 0.19(0.05,0.46) |  | 393.07(29.22,1653.45) | 2.70(2.36,3.05) |
| Libya | 1.19(0.76,1.76) | 0.07(0.05,0.11) |  | 19.59(11.35,33.58) | 0.50(0.29,0.85) |  | 1553.29(763.94,3100.71) | 6.77(6.56,6.97) |
| Lithuania | 28.44(25.43,32.00) | 1.60(1.43,1.80) |  | 10.85(8.79,12.84) | 0.89(0.72,1.05) |  | -61.84(-69.35,-52.94) | -2.73(-3.04,-2.41) |
| Luxembourg | 1.34(1.20,1.48) | 0.67(0.60,0.74) |  | 1.75(1.49,2.04) | 0.53(0.45,0.61) |  | 30.78(7.59,58.67) | -0.22(-0.49,0.04) |
| Madagascar | 0.49(0.12,0.92) | 0.01(0.00,0.02) |  | 2.04(0.53,4.05) | 0.02(0.00,0.03) |  | 318.03(128.63,686.07) | 1.21(1.08,1.34) |
| Malawi | 0.31(0.06,0.61) | 0.01(0.00,0.02) |  | 1.27(0.30,2.38) | 0.02(0.00,0.03) |  | 305.69(150.38,729.74) | 1.94(1.79,2.10) |
| Malaysia | 0.81(0.37,1.41) | 0.01(0.00,0.02) |  | 3.21(2.12,4.74) | 0.02(0.01,0.03) |  | 298.23(99.12,722.48) | 2.24(1.92,2.55) |
| Maldives | 0.00(0.00,0.00) | 0.00(0.00,0.01) |  | 0.02(0.00,0.03) | 0.00(0.00,0.01) |  | 614.17(200.23,1492.40) | 1.65(0.88,2.43) |
| Mali | 3.85(0.76,10.19) | 0.13(0.02,0.33) |  | 19.56(4.38,47.65) | 0.23(0.05,0.57) |  | 408.53(36.64,1661.99) | 1.89(1.77,2.00) |
| Malta | 0.36(0.33,0.40) | 0.20(0.18,0.22) |  | 0.59(0.51,0.69) | 0.30(0.25,0.34) |  | 62.30(34.45,95.76) | 1.41(1.28,1.54) |
| Marshall Islands | 0.00(0.00,0.00) | 0.00(0.00,0.00) |  | 0.00(0.00,0.00) | 0.00(0.00,0.00) |  | 162.56(45.31,543.63) | 1.14(1.06,1.22) |
| Mauritania | 1.09(0.37,2.68) | 0.14(0.05,0.35) |  | 4.08(1.30,9.92) | 0.24(0.08,0.58) |  | 273.58(-6.04,1349.59) | 1.30(1.16,1.43) |
| Mauritius | 0.00(0.00,0.00) | 0.00(0.00,0.00) |  | 0.43(0.37,0.48) | 0.07(0.06,0.07) |  | 445886.23(378640.77,518959.54) | 39.28(31.94,47.03) |
| Mexico | 34.28(33.20,35.53) | 0.10(0.09,0.10) |  | 178.26(153.79,204.86) | 0.28(0.24,0.32) |  | 420.08(340.45,504.80) | 3.55(3.15,3.96) |
| Micronesia (Federated States of) | 0.00(0.00,0.00) | 0.00(0.00,0.00) |  | 0.00(0.00,0.00) | 0.00(0.00,0.00) |  | 70.05(-12.52,234.75) | 0.93(0.86,1.01) |
| Monaco | 0.03(0.02,0.05) | 0.20(0.11,0.34) |  | 0.06(0.03,0.10) | 0.36(0.19,0.65) |  | 92.69(-12.96,303.17) | 2.02(1.68,2.35) |
| Mongolia | 0.79(0.24,1.73) | 0.09(0.03,0.20) |  | 2.35(1.02,4.79) | 0.14(0.06,0.29) |  | 198.85(-3.24,1021.64) | 1.73(1.56,1.89) |
| Montenegro | 2.23(1.37,3.66) | 0.74(0.45,1.21) |  | 2.09(1.29,3.06) | 0.71(0.44,1.04) |  | -6.21(-51.05,77.34) | 0.20(0.01,0.40) |
| Morocco | 6.24(2.26,10.96) | 0.06(0.02,0.10) |  | 43.28(23.37,74.80) | 0.24(0.13,0.41) |  | 593.29(252.63,1654.44) | 4.42(3.98,4.85) |
| Mozambique | 0.50(0.06,0.99) | 0.01(0.00,0.02) |  | 2.10(0.37,4.52) | 0.02(0.00,0.04) |  | 321.47(129.87,931.83) | 1.99(1.92,2.06) |
| Myanmar | 0.78(0.29,1.63) | 0.00(0.00,0.01) |  | 2.57(1.08,4.59) | 0.01(0.00,0.02) |  | 227.65(64.62,538.10) | 2.08(1.71,2.46) |
| Namibia | 0.03(0.01,0.06) | 0.01(0.00,0.01) |  | 0.13(0.06,0.23) | 0.01(0.01,0.02) |  | 287.65(99.70,682.69) | 1.79(1.65,1.94) |
| Nauru | 0.00(0.00,0.00) | 0.00(0.00,0.00) |  | 0.00(0.00,0.00) | 0.00(0.00,0.00) |  | 23.62(-33.15,139.90) | -0.01(-0.17,0.15) |
| Nepal | 0.48(0.09,0.98) | 0.01(0.00,0.01) |  | 1.89(0.53,3.46) | 0.01(0.00,0.02) |  | 294.96(124.94,741.34) | 2.50(2.34,2.67) |
| Netherlands | 49.10(44.95,53.10) | 0.63(0.58,0.68) |  | 56.66(50.76,62.57) | 0.74(0.66,0.82) |  | 15.40(2.35,31.55) | 0.58(0.29,0.88) |
| New Zealand | 5.97(5.18,6.89) | 0.36(0.31,0.42) |  | 9.37(7.87,11.12) | 0.38(0.32,0.46) |  | 57.00(24.71,96.18) | -0.15(-0.66,0.37) |
| Nicaragua | 0.61(0.43,0.81) | 0.04(0.03,0.06) |  | 2.72(1.84,3.97) | 0.08(0.06,0.12) |  | 342.80(183.00,579.98) | 2.53(2.06,3.00) |
| Niger | 2.36(0.29,7.02) | 0.09(0.01,0.26) |  | 10.46(1.21,28.57) | 0.13(0.02,0.35) |  | 344.05(4.04,1719.64) | 1.12(0.83,1.41) |
| Nigeria | 30.73(14.32,53.38) | 0.09(0.04,0.16) |  | 153.74(81.96,273.65) | 0.17(0.09,0.31) |  | 400.37(154.84,969.28) | 1.99(1.67,2.32) |
| Niue | 0.00(0.00,0.00) | 0.00(0.00,0.00) |  | 0.00(0.00,0.00) | 0.00(0.00,0.00) |  | -0.37(-46.72,108.72) | 0.39(0.30,0.47) |
| North Macedonia | 5.61(4.03,7.38) | 0.58(0.42,0.76) |  | 5.60(3.82,8.11) | 0.49(0.34,0.71) |  | -0.27(-37.17,53.88) | -0.72(-0.94,-0.50) |
| Northern Mariana Islands | 0.00(0.00,0.00) | 0.00(0.00,0.00) |  | 0.00(0.00,0.00) | 0.00(0.00,0.00) |  | 54.15(-14.64,209.53) | 2.45(2.07,2.83) |
| Norway | 17.06(16.18,17.97) | 0.83(0.79,0.88) |  | 19.03(17.72,20.42) | 0.74(0.69,0.80) |  | 11.56(2.24,21.23) | -0.54(-1.07,-0.01) |
| Oman | 0.40(0.18,0.72) | 0.04(0.02,0.08) |  | 4.02(2.01,7.01) | 0.14(0.07,0.24) |  | 914.51(267.14,3242.98) | 3.64(3.01,4.28) |
| Pakistan | 3.74(1.02,6.54) | 0.01(0.00,0.02) |  | 18.30(9.16,29.44) | 0.02(0.01,0.03) |  | 389.47(178.04,1213.44) | 1.76(1.63,1.89) |
| Palau | 0.00(0.00,0.00) | 0.00(0.00,0.00) |  | 0.00(0.00,0.00) | 0.00(0.00,0.00) |  | 85.38(1.88,271.94) | 1.27(1.18,1.35) |
| Palestine | 0.67(0.33,1.16) | 0.09(0.05,0.16) |  | 5.46(3.74,7.66) | 0.24(0.16,0.33) |  | 712.32(301.14,1533.81) | 4.81(4.07,5.56) |
| Panama | 0.60(0.54,0.65) | 0.06(0.05,0.06) |  | 2.74(2.10,3.46) | 0.13(0.10,0.17) |  | 358.53(250.15,497.07) | 2.97(2.44,3.50) |
| Papua New Guinea | 0.00(0.00,0.00) | 0.00(0.00,0.00) |  | 0.00(0.00,0.00) | 0.00(0.00,0.00) |  | 238.17(75.00,595.30) | 0.46(0.26,0.66) |
| Paraguay | 0.48(0.36,0.63) | 0.03(0.02,0.04) |  | 2.35(1.63,3.34) | 0.07(0.05,0.10) |  | 385.18(210.84,664.53) | 1.99(1.37,2.62) |
| Peru | 2.72(1.83,3.85) | 0.03(0.02,0.04) |  | 11.85(7.41,18.87) | 0.07(0.04,0.10) |  | 335.13(163.04,628.42) | 3.29(2.89,3.69) |
| Philippines | 7.22(5.11,9.20) | 0.03(0.02,0.04) |  | 19.82(16.34,24.31) | 0.04(0.03,0.04) |  | 174.55(103.23,316.68) | 0.60(0.39,0.81) |
| Poland | 272.54(259.45,285.36) | 1.51(1.44,1.58) |  | 108.46(97.45,120.09) | 0.59(0.53,0.65) |  | -60.20(-64.69,-54.98) | -3.38(-3.62,-3.13) |
| Portugal | 13.67(12.51,14.90) | 0.29(0.27,0.32) |  | 16.16(14.51,18.07) | 0.34(0.30,0.38) |  | 18.17(3.04,37.08) | 0.84(0.71,0.97) |
| Puerto Rico | 5.01(4.59,5.51) | 0.30(0.27,0.33) |  | 5.36(4.21,6.79) | 0.36(0.29,0.46) |  | 6.89(-16.80,41.43) | 0.69(0.43,0.94) |
| Qatar | 0.02(0.01,0.04) | 0.01(0.00,0.01) |  | 0.46(0.29,0.73) | 0.02(0.01,0.03) |  | 2165.08(1001.49,5034.18) | 3.56(2.68,4.45) |
| Republic of Korea | 7.96(6.46,9.34) | 0.03(0.03,0.04) |  | 7.01(5.11,9.30) | 0.03(0.02,0.04) |  | -11.96(-37.35,20.46) | -0.65(-0.90,-0.39) |
| Republic of Moldova | 6.20(5.62,6.89) | 0.30(0.27,0.33) |  | 3.17(2.65,3.74) | 0.17(0.14,0.20) |  | -48.84(-58.19,-38.66) | -2.73(-3.07,-2.39) |
| Romania | 83.79(74.12,93.75) | 0.77(0.68,0.86) |  | 34.58(28.90,41.90) | 0.39(0.33,0.47) |  | -58.73(-66.57,-48.93) | -2.90(-3.15,-2.65) |
| Russian Federation | 558.44(536.38,590.35) | 0.75(0.72,0.80) |  | 316.95(285.66,346.67) | 0.46(0.42,0.51) |  | -43.24(-49.73,-36.13) | -3.03(-3.58,-2.49) |
| Rwanda | 0.28(0.13,0.46) | 0.01(0.01,0.02) |  | 0.85(0.32,1.45) | 0.01(0.01,0.03) |  | 207.01(59.78,436.69) | 0.37(0.10,0.65) |
| Saint Kitts and Nevis | 0.07(0.06,0.08) | 0.42(0.38,0.47) |  | 0.13(0.10,0.16) | 0.40(0.31,0.51) |  | 81.29(35.25,136.94) | 0.05(-0.41,0.51) |
| Saint Lucia | 0.09(0.08,0.09) | 0.16(0.14,0.17) |  | 0.23(0.18,0.28) | 0.24(0.19,0.30) |  | 165.19(106.06,231.73) | 1.96(1.69,2.24) |
| Saint Vincent and the Grenadines | 0.04(0.03,0.04) | 0.08(0.07,0.10) |  | 0.09(0.07,0.11) | 0.16(0.13,0.20) |  | 145.24(91.64,208.05) | 2.20(1.93,2.48) |
| Samoa | 0.00(0.00,0.00) | 0.00(0.00,0.00) |  | 0.00(0.00,0.00) | 0.00(0.00,0.00) |  | 88.93(3.00,319.55) | 1.19(1.10,1.28) |
| San Marino | 0.00(0.00,0.00) | 0.00(0.00,0.00) |  | 0.00(0.00,0.00) | 0.00(0.00,0.00) |  | 35.76(-49.54,210.09) | 1.80(1.35,2.25) |
| Sao Tome and Principe | 0.01(0.00,0.02) | 0.02(0.01,0.06) |  | 0.04(0.01,0.10) | 0.04(0.01,0.10) |  | 317.05(-32.31,2160.70) | 1.48(0.77,2.20) |
| Saudi Arabia | 1.64(0.67,2.93) | 0.02(0.01,0.04) |  | 23.39(14.86,35.81) | 0.10(0.06,0.15) |  | 1328.23(507.23,3562.45) | 5.37(4.82,5.92) |
| Senegal | 3.64(0.77,10.08) | 0.14(0.03,0.38) |  | 13.53(3.80,36.95) | 0.21(0.06,0.57) |  | 271.28(-20.55,1396.65) | 1.48(1.30,1.67) |
| Serbia | 42.65(27.96,65.63) | 0.92(0.60,1.41) |  | 35.38(23.66,49.87) | 0.83(0.56,1.18) |  | -17.05(-51.90,38.52) | -0.40(-0.60,-0.21) |
| Seychelles | 0.00(0.00,0.00) | 0.00(0.00,0.01) |  | 0.01(0.00,0.01) | 0.01(0.01,0.02) |  | 529.42(199.82,1149.17) | 4.83(3.70,5.97) |
| Sierra Leone | 1.20(0.25,3.77) | 0.07(0.02,0.23) |  | 6.31(1.80,15.97) | 0.17(0.05,0.43) |  | 425.07(10.56,2790.01) | 3.23(2.98,3.49) |
| Singapore | 0.23(0.21,0.26) | 0.01(0.01,0.01) |  | 0.27(0.23,0.30) | 0.01(0.01,0.01) |  | 13.59(-3.82,33.26) | -1.39(-1.90,-0.88) |
| Slovakia | 15.73(11.25,22.07) | 0.63(0.45,0.89) |  | 14.54(9.61,22.64) | 0.54(0.36,0.85) |  | -7.58(-46.32,61.46) | -0.66(-0.82,-0.50) |
| Slovenia | 12.48(11.31,13.85) | 1.27(1.15,1.40) |  | 5.02(4.04,6.09) | 0.54(0.43,0.65) |  | -59.76(-67.99,-49.84) | -3.02(-3.22,-2.82) |
| Solomon Islands | 0.00(0.00,0.00) | 0.00(0.00,0.00) |  | 0.00(0.00,0.00) | 0.00(0.00,0.00) |  | 268.12(81.11,1130.68) | 1.32(1.11,1.54) |
| Somalia | 0.17(0.02,0.37) | 0.01(0.00,0.01) |  | 0.53(0.07,1.15) | 0.01(0.00,0.01) |  | 204.50(62.03,432.24) | -0.26(-0.47,-0.05) |
| South Africa | 12.48(9.67,15.45) | 0.08(0.06,0.10) |  | 27.17(20.80,34.24) | 0.09(0.07,0.12) |  | 117.62(60.23,178.32) | 0.30(0.13,0.48) |
| South Sudan | 0.14(0.03,0.28) | 0.01(0.00,0.01) |  | 0.44(0.10,0.91) | 0.01(0.00,0.03) |  | 214.94(71.95,521.28) | 2.06(1.79,2.33) |
| Spain | 48.69(44.82,53.19) | 0.27(0.25,0.29) |  | 64.73(56.86,72.70) | 0.30(0.27,0.34) |  | 32.95(14.41,52.01) | 0.55(0.43,0.67) |
| Sri Lanka | 0.60(0.44,0.82) | 0.01(0.01,0.01) |  | 0.92(0.57,1.41) | 0.01(0.01,0.01) |  | 53.34(-10.59,156.56) | 0.16(-0.24,0.55) |
| Sudan | 2.94(1.51,5.16) | 0.04(0.02,0.07) |  | 23.12(13.38,37.11) | 0.12(0.07,0.20) |  | 685.83(277.91,1746.56) | 3.74(3.46,4.01) |
| Suriname | 0.14(0.09,0.19) | 0.08(0.05,0.11) |  | 0.39(0.24,0.59) | 0.14(0.09,0.21) |  | 178.23(66.83,357.32) | 2.24(2.01,2.47) |
| Sweden | 24.34(22.60,26.19) | 0.59(0.55,0.64) |  | 24.44(21.27,27.67) | 0.53(0.46,0.60) |  | 0.39(-15.36,16.12) | -0.07(-0.29,0.15) |
| Switzerland | 30.59(27.57,34.18) | 0.85(0.77,0.95) |  | 28.54(25.15,32.46) | 0.67(0.59,0.77) |  | -6.71(-22.69,12.35) | -0.42(-0.65,-0.19) |
| Syrian Arab Republic | 2.15(1.04,3.80) | 0.05(0.02,0.08) |  | 6.45(3.66,11.22) | 0.10(0.06,0.18) |  | 199.97(45.01,501.06) | 2.52(2.16,2.89) |
| Taiwan (Province of China) | 0.43(0.40,0.46) | 0.00(0.00,0.00) |  | 1.84(1.56,2.18) | 0.02(0.01,0.02) |  | 326.78(254.32,411.28) | -0.88(-2.77,1.05) |
| Tajikistan | 0.55(0.35,0.78) | 0.03(0.02,0.04) |  | 1.29(0.48,2.64) | 0.03(0.01,0.06) |  | 135.62(-7.22,378.94) | 0.35(-0.49,1.19) |
| Thailand | 1.08(0.50,1.72) | 0.00(0.00,0.01) |  | 3.29(1.94,5.04) | 0.01(0.01,0.02) |  | 204.26(75.24,484.11) | 2.91(2.66,3.16) |
| Timor-Leste | 0.01(0.00,0.01) | 0.00(0.00,0.00) |  | 0.03(0.01,0.05) | 0.00(0.00,0.01) |  | 372.18(136.46,944.48) | 3.07(2.76,3.38) |
| Togo | 1.86(0.64,4.23) | 0.14(0.05,0.33) |  | 6.35(1.93,14.80) | 0.18(0.05,0.41) |  | 241.32(-13.20,1096.30) | 0.80(0.68,0.92) |
| Tokelau | 0.00(0.00,0.00) | 0.00(0.00,0.00) |  | 0.00(0.00,0.00) | 0.00(0.00,0.00) |  | 24.91(-33.03,146.34) | 0.60(0.46,0.73) |
| Tonga | 0.00(0.00,0.00) | 0.00(0.00,0.00) |  | 0.00(0.00,0.00) | 0.00(0.00,0.00) |  | 57.92(-15.01,266.02) | 0.90(0.79,1.01) |
| Trinidad and Tobago | 0.64(0.58,0.71) | 0.12(0.11,0.13) |  | 1.60(1.15,2.12) | 0.23(0.17,0.31) |  | 148.72(77.35,237.99) | 2.27(2.12,2.41) |
| Tunisia | 2.46(0.94,4.26) | 0.07(0.03,0.12) |  | 16.09(8.81,25.72) | 0.27(0.15,0.43) |  | 555.00(209.22,1417.67) | 4.32(3.82,4.82) |
| Turkey | 36.92(20.09,63.36) | 0.15(0.08,0.26) |  | 91.69(64.89,124.59) | 0.22(0.15,0.29) |  | 148.35(36.36,371.52) | 1.45(1.31,1.59) |
| Turkmenistan | 2.46(1.95,2.98) | 0.17(0.13,0.20) |  | 3.61(1.99,5.57) | 0.14(0.08,0.22) |  | 46.38(-19.75,129.30) | 0.02(-0.25,0.28) |
| Tuvalu | 0.00(0.00,0.00) | 0.00(0.00,0.00) |  | 0.00(0.00,0.00) | 0.00(0.00,0.00) |  | 61.90(-10.73,219.58) | 0.13(-0.08,0.34) |
| Uganda | 0.28(0.05,0.59) | 0.00(0.00,0.01) |  | 1.87(0.63,3.41) | 0.01(0.00,0.02) |  | 557.43(243.00,1900.12) | 2.62(2.43,2.81) |
| Ukraine | 261.78(240.76,282.36) | 1.04(0.96,1.12) |  | 151.59(103.32,205.52) | 0.72(0.49,0.98) |  | -42.09(-60.56,-20.79) | -2.69(-3.33,-2.05) |
| United Arab Emirates | 0.37(0.12,0.74) | 0.03(0.01,0.07) |  | 3.22(1.91,5.19) | 0.04(0.03,0.07) |  | 760.50(326.50,2240.68) | 0.21(-0.20,0.63) |
| United Kingdom | 264.03(258.67,269.64) | 0.96(0.94,0.98) |  | 340.32(325.84,352.80) | 1.09(1.05,1.13) |  | 28.90(23.14,34.36) | 0.86(0.64,1.08) |
| United Republic of Tanzania | 0.85(0.20,1.53) | 0.01(0.00,0.02) |  | 3.66(1.24,6.99) | 0.02(0.01,0.03) |  | 332.79(135.61,840.38) | 1.56(1.51,1.62) |
| United States of America | 650.87(632.17,672.68) | 0.51(0.50,0.53) |  | 757.44(722.91,791.99) | 0.50(0.48,0.52) |  | 16.37(9.82,23.72) | -0.40(-0.90,0.10) |
| United States Virgin Islands | 0.10(0.06,0.16) | 0.20(0.12,0.31) |  | 0.08(0.04,0.13) | 0.22(0.12,0.37) |  | -21.24(-58.94,43.30) | 0.94(0.67,1.20) |
| Uruguay | 5.63(5.18,6.11) | 0.41(0.37,0.44) |  | 4.96(4.24,5.56) | 0.31(0.27,0.35) |  | -11.96(-25.72,1.35) | -1.07(-1.28,-0.85) |
| Uzbekistan | 3.31(2.51,4.20) | 0.04(0.03,0.05) |  | 6.12(4.62,7.73) | 0.04(0.03,0.05) |  | 85.13(29.24,170.73) | 1.35(0.51,2.20) |
| Vanuatu | 0.00(0.00,0.00) | 0.00(0.00,0.00) |  | 0.00(0.00,0.00) | 0.00(0.00,0.00) |  | 223.10(68.59,736.04) | 0.65(0.49,0.80) |
| Venezuela (Bolivarian Republic of) | 7.86(7.25,8.56) | 0.10(0.09,0.10) |  | 37.30(27.43,48.88) | 0.29(0.22,0.39) |  | 374.73(251.67,535.05) | 3.46(3.10,3.82) |
| Viet Nam | 1.08(0.29,2.10) | 0.00(0.00,0.01) |  | 5.24(2.07,9.18) | 0.01(0.00,0.02) |  | 386.36(147.13,981.66) | 3.00(2.72,3.29) |
| Yemen | 1.32(0.42,2.65) | 0.03(0.01,0.06) |  | 12.84(7.17,23.82) | 0.09(0.05,0.17) |  | 873.75(381.65,2724.19) | 3.56(3.18,3.95) |
| Zambia | 0.35(0.11,0.67) | 0.01(0.00,0.02) |  | 1.74(0.76,3.11) | 0.02(0.01,0.04) |  | 397.87(123.80,1079.53) | 1.59(1.42,1.76) |
| Zimbabwe | 0.00(0.00,0.00) | 0.00(0.00,0.00) |  | 0.00(0.00,0.00) | 0.00(0.00,0.00) |  | 249.94(87.54,648.06) | 2.25(1.81,2.69) |
