# Supplementary material for: Emerging epidemiological trends of multiple sclerosis among adults aged 20–54 years, 1990–2021, with projections to 2035: a systematic analysis for the global burden of disease study 2021
Source: Front Neurol. 2025 Jul 10;16:1616245. doi: 10.3389/fneur.2025.1616245 (PMC12286822; doi:10.3389/fneur.2025.1616245)
Supplement: Supplementary file 4 [file Table_4.DOCX]

Table S4. DALYs of multiple sclerosis Between 1990 and 2021 at the Global and Regional Levels

| location | 1990 | |  | 2021 | |  | 1990-2021 | |
| --- | --- | --- | --- | --- | --- | --- | --- | --- |
|  | DALYs cases | DALY rate |  | DALYs cases | DALY rate |  | Cases change | EAPC |
| Afghanistan | 526.52(346.70,748.26) | 16.69(10.99,23.71) |  | 3036.25(2102.12,4268.91) | 24.85(17.21,34.94) |  | 476.66(336.27,661.87) | 1.85(1.60,2.09) |
| Albania | 1184.57(883.03,1531.76) | 78.33(58.39,101.29) |  | 963.11(675.50,1418.97) | 76.17(53.42,112.22) |  | -18.70(-48.21,28.50) | 0.03(-0.19,0.25) |
| Algeria | 1327.94(946.55,1842.21) | 13.72(9.78,19.04) |  | 6413.29(4839.26,8331.60) | 29.59(22.33,38.44) |  | 382.95(269.54,520.92) | 2.49(2.40,2.59) |
| American Samoa | 0.17(0.10,0.26) | 0.81(0.50,1.22) |  | 0.21(0.13,0.31) | 0.94(0.59,1.38) |  | 23.46(14.90,33.48) | 0.49(0.47,0.51) |
| Andorra | 14.53(10.64,20.33) | 46.82(34.29,65.51) |  | 29.01(20.01,41.54) | 64.81(44.72,92.82) |  | 99.62(32.87,194.70) | 1.47(1.28,1.65) |
| Angola | 89.95(61.65,133.53) | 2.31(1.59,3.44) |  | 355.56(248.08,504.91) | 2.94(2.05,4.17) |  | 295.31(222.78,396.20) | 0.80(0.73,0.88) |
| Antigua and Barbuda | 4.35(3.72,5.01) | 15.71(13.45,18.10) |  | 10.52(8.77,12.36) | 22.31(18.58,26.21) |  | 141.81(104.78,183.51) | 1.78(1.55,2.01) |
| Argentina | 3230.32(2737.85,3896.98) | 22.25(18.86,26.84) |  | 3762.87(3053.28,4611.83) | 16.77(13.60,20.55) |  | 16.49(0.38,32.14) | -0.94(-1.07,-0.82) |
| Armenia | 151.70(115.99,195.85) | 9.45(7.22,12.19) |  | 170.28(124.42,219.94) | 11.78(8.61,15.22) |  | 12.25(-4.76,33.45) | 1.37(1.06,1.69) |
| Australia | 2347.05(1925.10,2865.63) | 27.92(22.90,34.09) |  | 4844.46(3829.74,5997.34) | 39.89(31.53,49.38) |  | 106.41(75.81,140.12) | 1.18(0.79,1.57) |
| Austria | 1868.19(1558.67,2256.47) | 47.31(39.47,57.14) |  | 2513.29(2002.79,2997.57) | 58.83(46.88,70.16) |  | 34.53(16.14,54.86) | 1.05(0.90,1.20) |
| Azerbaijan | 222.26(158.66,301.85) | 6.69(4.77,9.08) |  | 416.21(286.69,579.25) | 7.52(5.18,10.46) |  | 87.26(45.35,145.37) | 0.61(0.48,0.73) |
| Bahamas | 22.24(19.69,24.88) | 17.87(15.82,19.99) |  | 62.40(48.92,77.78) | 31.09(24.37,38.75) |  | 180.54(116.67,251.15) | 2.10(1.94,2.26) |
| Bahrain | 23.30(15.35,32.94) | 8.37(5.52,11.83) |  | 162.51(117.33,216.84) | 16.86(12.18,22.50) |  | 597.32(456.58,800.15) | 2.34(2.16,2.53) |
| Bangladesh | 1192.86(837.54,1674.35) | 2.90(2.03,4.07) |  | 3070.80(2183.29,4238.82) | 3.83(2.72,5.28) |  | 157.43(130.32,197.65) | 0.94(0.85,1.03) |
| Barbados | 24.91(22.07,28.08) | 20.48(18.14,23.09) |  | 45.58(35.76,56.54) | 32.21(25.27,39.95) |  | 82.95(43.26,125.66) | 1.85(1.62,2.09) |
| Belarus | 1742.94(1571.83,1946.37) | 34.92(31.49,38.99) |  | 1319.14(1058.99,1596.12) | 29.83(23.94,36.09) |  | -24.31(-39.30,-7.80) | -1.25(-1.71,-0.79) |
| Belgium | 2469.14(2060.79,2897.66) | 50.54(42.18,59.31) |  | 3258.62(2639.91,3886.68) | 63.34(51.32,75.55) |  | 31.97(14.37,48.86) | 0.68(0.50,0.86) |
| Belize | 3.29(2.65,4.04) | 4.78(3.85,5.86) |  | 18.41(15.51,22.14) | 8.77(7.39,10.55) |  | 458.94(388.29,544.63) | 2.21(1.98,2.44) |
| Benin | 153.07(65.80,331.90) | 9.22(3.96,19.98) |  | 724.57(307.00,1610.76) | 14.05(5.95,31.24) |  | 373.35(74.97,1208.53) | 1.14(1.02,1.26) |
| Bermuda | 5.63(4.96,6.39) | 17.12(15.08,19.44) |  | 5.40(4.45,6.51) | 18.79(15.48,22.65) |  | -4.08(-20.19,14.61) | 0.36(0.25,0.47) |
| Bhutan | 7.32(5.12,10.33) | 2.93(2.05,4.14) |  | 16.32(11.56,22.43) | 4.06(2.87,5.58) |  | 122.87(101.93,158.19) | 0.94(0.87,1.01) |
| Bolivia (Plurinational State of) | 128.32(85.23,189.89) | 5.12(3.40,7.57) |  | 478.00(314.73,731.34) | 8.38(5.52,12.82) |  | 272.50(157.89,464.82) | 1.59(1.58,1.60) |
| Bosnia and Herzegovina | 833.30(645.58,1062.72) | 36.50(28.28,46.55) |  | 443.79(321.00,598.27) | 28.77(20.81,38.79) |  | -46.74(-62.51,-21.11) | -0.99(-1.19,-0.79) |
| Botswana | 11.98(8.28,16.51) | 2.47(1.71,3.40) |  | 36.57(25.32,49.83) | 2.96(2.05,4.04) |  | 205.12(171.46,243.28) | 0.59(0.48,0.70) |
| Brazil | 5417.07(4289.86,6826.45) | 8.14(6.45,10.26) |  | 13105.05(10585.24,16269.95) | 11.58(9.36,14.38) |  | 141.92(127.16,158.67) | 0.92(0.65,1.19) |
| Brunei Darussalam | 3.28(2.13,5.10) | 2.55(1.66,3.97) |  | 8.25(5.14,12.91) | 3.15(1.96,4.93) |  | 151.95(102.10,238.28) | 0.74(0.71,0.77) |
| Bulgaria | 2349.02(2085.48,2653.02) | 57.95(51.45,65.45) |  | 1828.78(1538.97,2179.23) | 58.70(49.39,69.94) |  | -22.15(-34.70,-6.58) | -0.14(-0.29,0.00) |
| Burkina Faso | 288.63(114.17,648.88) | 9.17(3.63,20.61) |  | 1149.07(438.24,2870.80) | 13.60(5.19,33.97) |  | 298.11(42.73,1043.38) | 1.37(1.17,1.56) |
| Burundi | 38.27(26.25,55.17) | 1.89(1.30,2.73) |  | 103.22(69.82,144.92) | 2.01(1.36,2.82) |  | 169.73(135.98,221.60) | -0.11(-0.25,0.02) |
| Cabo Verde | 16.96(5.77,38.91) | 14.15(4.81,32.46) |  | 32.92(14.45,74.10) | 11.36(4.98,25.56) |  | 94.04(-28.05,454.92) | -0.28(-0.61,0.04) |
| Cambodia | 39.61(26.74,58.31) | 1.03(0.70,1.52) |  | 111.45(77.27,158.57) | 1.35(0.94,1.92) |  | 181.38(142.07,248.53) | 0.79(0.75,0.83) |
| Cameroon | 424.69(199.51,848.40) | 11.30(5.31,22.57) |  | 2065.91(817.66,4837.93) | 16.09(6.37,37.67) |  | 386.45(47.25,1454.07) | 1.13(1.09,1.16) |
| Canada | 9471.39(7733.51,11318.69) | 67.03(54.73,80.11) |  | 13378.15(10656.77,16071.39) | 78.84(62.80,94.71) |  | 41.25(25.74,57.52) | 0.35(0.13,0.56) |
| Central African Republic | 21.27(15.01,30.70) | 2.04(1.44,2.94) |  | 51.18(35.67,71.95) | 2.29(1.60,3.22) |  | 140.57(107.54,188.62) | 0.36(0.29,0.43) |
| Chad | 155.11(66.02,351.70) | 7.61(3.24,17.26) |  | 719.23(274.80,1673.09) | 12.25(4.68,28.50) |  | 363.70(66.16,1097.05) | 1.63(1.56,1.70) |
| Chile | 810.91(657.47,1004.10) | 12.81(10.39,15.86) |  | 1061.07(812.41,1353.90) | 11.26(8.62,14.36) |  | 30.85(13.18,51.11) | -0.40(-0.49,-0.30) |
| China | 5306.18(3706.29,7578.72) | 0.90(0.63,1.29) |  | 9457.84(6702.80,12728.15) | 1.33(0.94,1.79) |  | 78.24(51.56,102.82) | 1.08(0.97,1.19) |
| Colombia | 829.12(732.23,945.85) | 5.69(5.03,6.50) |  | 2218.43(1831.65,2608.07) | 8.90(7.34,10.46) |  | 167.56(128.17,209.99) | 1.55(1.16,1.94) |
| Comoros | 4.13(2.72,5.84) | 2.49(1.64,3.52) |  | 11.19(7.73,15.56) | 3.20(2.21,4.45) |  | 171.14(126.12,235.86) | 0.71(0.60,0.83) |
| Congo | 18.38(12.86,25.06) | 2.05(1.43,2.79) |  | 69.98(49.49,92.83) | 2.83(2.00,3.76) |  | 280.68(200.86,395.28) | 1.07(0.99,1.14) |
| Cook Islands | 0.08(0.05,0.12) | 1.00(0.62,1.49) |  | 0.09(0.06,0.13) | 1.13(0.72,1.63) |  | 8.25(1.38,15.58) | 0.43(0.40,0.47) |
| Costa Rica | 85.58(73.52,100.32) | 6.39(5.49,7.49) |  | 353.14(302.05,407.72) | 14.58(12.47,16.84) |  | 312.66(257.29,368.18) | 2.53(2.25,2.82) |
| Croatia | 428.87(180.82,971.91) | 9.25(3.90,20.95) |  | 1581.96(651.69,3856.19) | 13.54(5.58,33.00) |  | -40.55(-49.90,-29.50) | 1.52(1.42,1.63) |
| Cuba | 1119.66(995.15,1262.11) | 46.23(41.09,52.11) |  | 665.62(554.03,795.28) | 34.82(28.98,41.60) |  | 33.51(13.85,54.86) | -0.84(-0.93,-0.75) |
| Cyprus | 914.00(823.92,1031.90) | 16.62(14.98,18.76) |  | 1220.29(1025.73,1433.70) | 22.39(18.82,26.30) |  | 163.17(78.52,294.31) | 1.18(1.05,1.32) |
| Czechia | 91.71(61.56,136.51) | 24.18(16.23,35.99) |  | 241.35(175.74,318.21) | 33.37(24.30,44.00) |  | -41.44(-51.36,-30.79) | 1.11(1.02,1.20) |
| C么te d'Ivoire | 3595.48(3229.64,4004.87) | 73.64(66.14,82.02) |  | 2105.36(1699.75,2523.79) | 42.81(34.56,51.31) |  | 268.86(22.55,989.03) | -1.90(-2.10,-1.70) |
| Democratic People's Republic of Korea | 167.01(117.33,241.11) | 1.65(1.16,2.38) |  | 284.17(201.28,401.78) | 2.01(1.42,2.84) |  | 70.15(47.96,101.51) | 0.66(0.63,0.68) |
| Democratic Republic of the Congo | 256.96(180.56,371.75) | 1.85(1.30,2.68) |  | 783.22(553.64,1081.26) | 2.17(1.53,3.00) |  | 204.80(162.04,263.72) | 0.45(0.34,0.57) |
| Denmark | 2429.89(2099.56,2799.40) | 93.86(81.10,108.13) |  | 2259.98(1870.71,2667.93) | 85.85(71.07,101.35) |  | -6.99(-18.83,5.24) | -0.18(-0.38,0.01) |
| Djibouti | 3.83(2.61,5.53) | 2.27(1.55,3.28) |  | 19.11(12.86,27.44) | 3.05(2.05,4.38) |  | 399.12(322.71,542.39) | 0.89(0.81,0.97) |
| Dominica | 1.63(1.26,2.10) | 5.46(4.23,7.04) |  | 2.98(2.13,4.21) | 9.14(6.53,12.94) |  | 82.68(34.69,144.09) | 2.01(1.84,2.17) |
| Dominican Republic | 159.10(124.53,200.95) | 5.24(4.10,6.62) |  | 411.41(304.53,542.68) | 7.55(5.59,9.96) |  | 158.59(95.07,259.71) | 1.55(1.40,1.70) |
| Ecuador | 175.81(150.43,208.81) | 4.23(3.62,5.02) |  | 600.59(481.78,735.68) | 6.92(5.55,8.47) |  | 241.62(189.92,311.45) | 2.45(1.90,3.00) |
| Egypt | 1422.67(958.25,1953.56) | 6.20(4.18,8.52) |  | 6295.04(4105.91,9088.28) | 13.11(8.55,18.93) |  | 342.48(267.52,441.21) | 2.53(2.37,2.69) |
| El Salvador | 78.55(63.77,98.14) | 3.81(3.09,4.76) |  | 227.09(174.84,292.18) | 7.46(5.74,9.59) |  | 189.10(128.70,284.27) | 2.43(2.20,2.66) |
| Equatorial Guinea | 2.92(2.01,4.24) | 1.93(1.33,2.81) |  | 16.38(11.58,22.80) | 2.47(1.75,3.44) |  | 461.41(321.98,718.49) | 0.84(0.79,0.88) |
| Eritrea | 32.70(22.52,47.27) | 2.59(1.78,3.74) |  | 95.03(63.21,136.18) | 3.23(2.15,4.63) |  | 190.61(138.17,273.56) | 0.69(0.65,0.74) |
| Estonia | 602.71(549.65,656.25) | 80.29(73.22,87.42) |  | 191.54(161.17,222.05) | 32.30(27.18,37.44) |  | -68.22(-73.81,-62.50) | -4.31(-4.78,-3.84) |
| Eswatini | 7.59(5.19,10.53) | 2.67(1.83,3.71) |  | 16.59(11.75,22.46) | 3.13(2.22,4.24) |  | 118.65(93.73,154.66) | 0.44(0.38,0.51) |
| Ethiopia | 405.64(279.94,591.06) | 2.27(1.56,3.30) |  | 1059.12(697.11,1462.14) | 2.36(1.55,3.26) |  | 161.10(127.00,213.70) | -0.05(-0.18,0.09) |
| Fiji | 3.08(1.87,4.61) | 0.90(0.55,1.34) |  | 4.25(2.64,6.29) | 0.97(0.60,1.44) |  | 37.91(30.41,47.38) | 0.19(0.13,0.25) |
| Finland | 1471.79(1256.09,1699.13) | 57.63(49.18,66.53) |  | 1418.49(1179.91,1685.28) | 59.73(49.69,70.97) |  | -3.62(-14.43,8.68) | 0.07(-0.07,0.21) |
| France | 12548.61(10244.84,15455.08) | 45.28(36.97,55.77) |  | 16360.85(12967.27,19699.89) | 57.35(45.45,69.05) |  | 30.38(14.24,47.76) | 0.84(0.58,1.10) |
| Gabon | 7.71(5.64,10.42) | 2.04(1.50,2.76) |  | 22.62(16.16,30.86) | 2.79(1.99,3.81) |  | 193.58(126.92,299.74) | 0.97(0.90,1.04) |
| Gambia | 35.12(13.76,82.13) | 9.64(3.78,22.54) |  | 190.86(69.06,429.68) | 19.62(7.10,44.17) |  | 443.50(61.05,1510.26) | 2.18(1.95,2.42) |
| Georgia | 206.33(153.66,275.64) | 7.89(5.88,10.54) |  | 159.01(116.67,211.82) | 9.77(7.17,13.01) |  | -22.94(-36.10,-7.54) | 1.39(1.07,1.71) |
| Germany | 25226.17(21384.04,29858.70) | 60.60(51.37,71.73) |  | 23665.23(19454.48,28679.26) | 62.42(51.32,75.65) |  | -6.19(-17.17,5.25) | 0.36(0.21,0.52) |
| Ghana | 930.60(436.08,1865.79) | 16.35(7.66,32.79) |  | 3414.54(1692.30,7097.59) | 22.46(11.13,46.68) |  | 266.92(39.91,933.72) | 1.07(0.99,1.15) |
| Greece | 1186.79(1012.78,1391.67) | 24.20(20.65,28.37) |  | 2038.14(1747.58,2373.57) | 44.36(38.04,51.67) |  | 71.74(51.04,94.07) | 2.22(2.15,2.29) |
| Greenland | 8.37(5.83,11.04) | 26.03(18.12,34.33) |  | 8.87(6.58,11.69) | 32.66(24.22,43.05) |  | 5.93(-13.35,35.65) | 1.34(1.06,1.62) |
| Grenada | 5.90(5.22,6.67) | 18.00(15.93,20.33) |  | 11.58(9.61,13.86) | 22.08(18.32,26.42) |  | 96.09(61.95,136.34) | 1.20(0.92,1.48) |
| Guam | 0.56(0.34,0.84) | 0.81(0.49,1.21) |  | 0.67(0.41,0.97) | 0.91(0.57,1.33) |  | 18.39(11.94,26.40) | 0.36(0.29,0.43) |
| Guatemala | 151.23(131.11,176.09) | 5.22(4.52,6.08) |  | 585.23(488.59,703.58) | 7.99(6.67,9.61) |  | 286.98(239.04,341.25) | 1.41(1.10,1.71) |
| Guinea | 169.79(77.93,363.25) | 7.88(3.62,16.87) |  | 759.98(316.89,1746.59) | 15.04(6.27,34.57) |  | 347.61(71.73,1004.63) | 2.20(2.12,2.28) |
| Guinea-Bissau | 44.53(19.51,96.05) | 12.54(5.49,27.04) |  | 151.07(67.39,340.14) | 18.19(8.11,40.96) |  | 239.23(17.96,792.78) | 1.21(1.07,1.36) |
| Guyana | 15.68(13.04,18.74) | 4.65(3.87,5.56) |  | 31.44(23.49,40.70) | 8.47(6.33,10.96) |  | 100.58(51.30,161.96) | 2.35(2.08,2.61) |
| Haiti | 203.96(130.49,320.06) | 8.17(5.23,12.83) |  | 673.51(417.30,1106.10) | 11.09(6.87,18.22) |  | 230.22(116.04,395.57) | 1.01(0.95,1.06) |
| Honduras | 43.67(31.88,59.23) | 2.63(1.92,3.56) |  | 161.27(112.68,222.65) | 3.43(2.39,4.73) |  | 269.26(197.84,366.85) | 0.79(0.74,0.84) |
| Hungary | 3411.52(3049.01,3832.54) | 69.38(62.01,77.94) |  | 1973.81(1660.23,2326.49) | 43.46(36.55,51.22) |  | -42.14(-50.51,-30.99) | -1.81(-1.91,-1.70) |
| Iceland | 72.82(60.08,85.92) | 59.85(49.38,70.62) |  | 119.03(97.31,142.31) | 72.62(59.37,86.83) |  | 63.46(40.19,86.59) | 0.73(0.68,0.78) |
| India | 11315.21(8024.04,15734.52) | 3.09(2.19,4.30) |  | 28868.78(20902.37,38524.51) | 4.05(2.93,5.40) |  | 155.13(134.35,176.84) | 0.91(0.87,0.96) |
| Indonesia | 814.52(560.64,1150.30) | 1.00(0.69,1.41) |  | 1963.88(1413.03,2640.22) | 1.34(0.96,1.80) |  | 141.11(112.09,186.59) | 0.91(0.86,0.96) |
| Iran (Islamic Republic of) | 5845.11(4238.54,7644.62) | 27.86(20.20,36.44) |  | 18701.50(15499.57,22269.49) | 40.21(33.32,47.88) |  | 219.95(159.16,310.96) | 1.65(1.52,1.79) |
| Iraq | 821.66(586.29,1141.64) | 11.98(8.55,16.65) |  | 3573.66(2522.34,4815.20) | 18.14(12.80,24.44) |  | 334.93(247.80,450.04) | 1.43(1.35,1.51) |
| Ireland | 1166.93(949.38,1392.63) | 73.27(59.61,87.44) |  | 1982.19(1585.84,2382.27) | 85.91(68.73,103.24) |  | 69.86(48.46,92.97) | 0.74(0.63,0.84) |
| Israel | 396.42(311.33,495.79) | 18.49(14.52,23.12) |  | 927.15(727.09,1160.01) | 21.96(17.22,27.48) |  | 133.88(102.75,175.09) | 0.67(0.57,0.77) |
| Italy | 11750.80(9700.49,14125.29) | 42.03(34.69,50.52) |  | 14490.88(11661.67,17691.97) | 54.45(43.82,66.47) |  | 23.32(15.44,30.01) | 1.18(1.05,1.30) |
| Jamaica | 55.35(45.53,68.28) | 5.66(4.65,6.98) |  | 157.44(122.05,203.60) | 10.82(8.38,13.99) |  | 184.47(128.94,260.80) | 2.19(1.87,2.51) |
| Japan | 3230.06(2456.88,4209.14) | 5.14(3.91,6.70) |  | 3157.75(2409.57,4068.32) | 5.82(4.44,7.50) |  | -2.24(-5.49,0.94) | 0.48(0.38,0.57) |
| Jordan | 348.78(266.77,448.10) | 24.49(18.73,31.47) |  | 1781.00(1367.10,2306.95) | 29.00(22.26,37.56) |  | 410.63(295.67,570.68) | 0.49(0.34,0.64) |
| Kazakhstan | 1967.01(1617.05,2348.79) | 25.68(21.11,30.67) |  | 1926.98(1382.41,2506.98) | 21.27(15.26,27.68) |  | -2.04(-25.31,23.10) | -0.81(-0.96,-0.65) |
| Kenya | 141.78(99.46,198.54) | 1.77(1.24,2.47) |  | 529.81(386.66,693.26) | 2.44(1.78,3.19) |  | 273.68(230.70,334.47) | 1.01(0.95,1.06) |
| Kiribati | 0.22(0.13,0.32) | 0.68(0.41,1.01) |  | 0.43(0.26,0.63) | 0.78(0.48,1.14) |  | 98.70(87.54,111.42) | 0.31(0.24,0.39) |
| Kuwait | 96.05(62.12,137.36) | 10.21(6.60,14.60) |  | 740.53(521.53,1015.50) | 23.98(16.89,32.88) |  | 670.99(521.86,864.39) | 2.94(2.66,3.21) |
| Kyrgyzstan | 145.75(114.34,182.78) | 7.93(6.22,9.95) |  | 245.07(184.86,315.39) | 7.67(5.79,9.87) |  | 68.15(43.68,95.87) | 0.35(-0.08,0.77) |
| Lao People's Democratic Republic | 17.82(12.00,26.36) | 1.14(0.77,1.69) |  | 52.13(35.73,75.53) | 1.44(0.99,2.09) |  | 192.50(152.53,263.86) | 0.67(0.63,0.72) |
| Latvia | 1172.87(1068.55,1287.44) | 91.60(83.45,100.55) |  | 416.36(350.78,486.15) | 50.45(42.51,58.91) |  | -64.50(-70.22,-58.00) | -3.19(-3.62,-2.76) |
| Lebanon | 226.73(162.80,315.42) | 17.80(12.78,24.76) |  | 791.20(596.84,1058.04) | 27.34(20.62,36.56) |  | 248.97(170.25,347.23) | 1.34(1.26,1.42) |
| Lesotho | 16.05(10.97,22.90) | 2.89(1.97,4.12) |  | 26.91(18.89,37.91) | 3.15(2.21,4.43) |  | 67.63(50.80,93.58) | 0.20(0.13,0.27) |
| Liberia | 78.74(36.08,171.62) | 8.69(3.98,18.94) |  | 351.88(141.53,807.67) | 15.04(6.05,34.53) |  | 346.89(55.63,1055.70) | 2.13(1.91,2.36) |
| Libya | 216.48(156.77,292.32) | 13.57(9.82,18.32) |  | 1639.33(1193.15,2315.57) | 41.54(30.23,58.67) |  | 657.27(444.90,1022.13) | 4.07(3.92,4.22) |
| Lithuania | 1471.94(1314.23,1641.11) | 82.96(74.07,92.50) |  | 597.35(495.58,692.20) | 48.77(40.46,56.52) |  | -59.42(-66.41,-51.63) | -2.48(-2.76,-2.19) |
| Luxembourg | 117.62(98.01,140.83) | 58.94(49.11,70.57) |  | 196.24(159.08,238.03) | 59.17(47.97,71.77) |  | 66.84(44.10,91.72) | 0.22(0.08,0.37) |
| Madagascar | 118.47(80.93,168.57) | 2.74(1.87,3.89) |  | 368.35(245.64,530.17) | 3.13(2.09,4.51) |  | 210.91(165.38,285.32) | 0.36(0.28,0.44) |
| Malawi | 81.52(55.10,117.87) | 2.26(1.53,3.27) |  | 211.20(134.01,312.17) | 2.75(1.74,4.06) |  | 159.07(128.31,217.42) | 0.59(0.48,0.70) |
| Malaysia | 100.11(67.34,140.90) | 1.28(0.86,1.80) |  | 299.35(223.05,392.75) | 1.80(1.34,2.36) |  | 199.03(114.94,305.94) | 1.17(1.03,1.31) |
| Maldives | 0.67(0.46,0.97) | 0.85(0.59,1.24) |  | 3.41(2.29,4.84) | 1.03(0.69,1.46) |  | 410.19(332.85,548.93) | 0.49(0.31,0.68) |
| Mali | 351.19(138.35,791.69) | 11.52(4.54,25.97) |  | 1578.72(549.22,3502.19) | 18.74(6.52,41.58) |  | 349.54(66.25,975.78) | 1.51(1.41,1.61) |
| Malta | 30.84(25.80,37.26) | 16.86(14.11,20.37) |  | 48.27(40.57,58.05) | 24.08(20.24,28.96) |  | 56.52(36.56,80.84) | 1.23(1.16,1.29) |
| Marshall Islands | 0.12(0.07,0.18) | 0.74(0.45,1.11) |  | 0.23(0.14,0.34) | 0.84(0.53,1.24) |  | 93.87(81.54,107.76) | 0.44(0.39,0.49) |
| Mauritania | 97.98(47.82,200.10) | 12.98(6.34,26.51) |  | 336.84(144.16,737.91) | 19.75(8.45,43.27) |  | 243.80(16.47,832.58) | 1.10(0.99,1.20) |
| Mauritius | 5.92(3.69,8.86) | 1.09(0.68,1.63) |  | 29.12(25.05,33.74) | 4.57(3.93,5.30) |  | 392.24(267.38,624.51) | 6.04(5.40,6.69) |
| Mexico | 2678.39(2348.56,3086.09) | 7.64(6.70,8.80) |  | 11733.62(9974.74,13454.51) | 18.17(15.45,20.84) |  | 338.08(289.54,393.11) | 2.98(2.66,3.29) |
| Micronesia (Federated States of) | 0.27(0.16,0.41) | 0.71(0.43,1.09) |  | 0.38(0.24,0.56) | 0.79(0.49,1.16) |  | 39.96(32.36,47.99) | 0.31(0.30,0.32) |
| Monaco | 3.91(2.85,5.11) | 26.96(19.71,35.30) |  | 6.04(4.28,8.48) | 39.12(27.73,54.88) |  | 54.72(9.31,117.84) | 1.29(1.14,1.44) |
| Mongolia | 77.77(46.37,127.05) | 9.16(5.46,14.96) |  | 206.88(128.64,337.68) | 12.70(7.90,20.73) |  | 166.03(41.29,409.14) | 1.25(1.15,1.36) |
| Montenegro | 150.96(106.49,214.22) | 49.86(35.18,70.76) |  | 155.81(114.40,205.10) | 53.12(39.01,69.93) |  | 3.21(-34.30,60.81) | 0.39(0.25,0.53) |
| Morocco | 1421.99(992.63,1984.22) | 13.50(9.42,18.84) |  | 5299.65(3855.15,7499.48) | 29.03(21.12,41.09) |  | 272.69(181.20,416.79) | 2.53(2.42,2.63) |
| Mozambique | 129.94(86.26,188.71) | 2.70(1.79,3.92) |  | 355.16(222.83,523.12) | 3.08(1.93,4.54) |  | 173.33(134.72,238.02) | 0.48(0.39,0.57) |
| Myanmar | 227.11(157.40,335.56) | 1.30(0.90,1.93) |  | 455.22(325.76,647.06) | 1.67(1.20,2.38) |  | 100.44(69.56,149.61) | 0.72(0.65,0.80) |
| Namibia | 12.68(8.81,17.72) | 2.38(1.65,3.32) |  | 32.61(23.15,44.97) | 2.88(2.04,3.97) |  | 157.09(128.16,198.67) | 0.58(0.49,0.67) |
| Nauru | 0.03(0.02,0.04) | 0.64(0.39,0.97) |  | 0.03(0.02,0.05) | 0.68(0.41,1.03) |  | 23.53(18.46,31.03) | 0.17(0.14,0.20) |
| Nepal | 245.69(166.25,349.02) | 3.24(2.19,4.60) |  | 604.70(437.00,842.17) | 4.11(2.97,5.72) |  | 146.12(123.00,175.46) | 0.86(0.79,0.94) |
| Netherlands | 4585.23(3823.09,5451.57) | 58.95(49.15,70.09) |  | 5383.47(4410.45,6447.35) | 70.20(57.51,84.07) |  | 17.41(3.53,34.58) | 0.60(0.40,0.80) |
| New Zealand | 498.30(411.69,599.20) | 30.05(24.83,36.14) |  | 740.83(607.36,872.12) | 30.37(24.90,35.75) |  | 48.67(28.43,74.36) | -0.19(-0.52,0.15) |
| Nicaragua | 57.61(45.29,72.21) | 4.15(3.26,5.20) |  | 238.65(180.36,315.26) | 7.33(5.54,9.68) |  | 314.28(224.11,427.00) | 2.15(1.84,2.46) |
| Niger | 234.18(85.40,546.46) | 8.54(3.12,19.94) |  | 932.41(295.49,2172.56) | 11.57(3.67,26.96) |  | 298.17(54.78,1031.89) | 0.85(0.64,1.05) |
| Nigeria | 2804.65(1697.48,4231.50) | 8.19(4.96,12.36) |  | 12738.92(7709.36,20897.42) | 14.41(8.72,23.64) |  | 354.21(167.51,716.68) | 1.74(1.49,2.00) |
| Niue | 0.01(0.01,0.01) | 1.05(0.64,1.55) |  | 0.01(0.01,0.01) | 1.14(0.72,1.66) |  | -11.98(-16.44,-6.73) | 0.24(0.20,0.28) |
| North Macedonia | 375.72(289.29,479.35) | 38.88(29.93,49.60) |  | 454.55(346.28,573.89) | 39.98(30.46,50.48) |  | 20.98(-8.88,60.25) | -0.03(-0.18,0.12) |
| Northern Mariana Islands | 0.24(0.15,0.36) | 0.92(0.56,1.36) |  | 0.26(0.16,0.38) | 1.08(0.68,1.58) |  | 5.57(-2.23,14.85) | 0.72(0.59,0.85) |
| Norway | 1392.14(1183.27,1619.00) | 67.89(57.70,78.95) |  | 2098.89(1694.83,2542.67) | 82.15(66.34,99.52) |  | 50.77(38.40,63.13) | 0.46(0.18,0.74) |
| Oman | 98.67(70.03,136.76) | 11.19(7.94,15.51) |  | 659.32(459.68,894.74) | 22.74(15.85,30.86) |  | 568.23(384.94,833.05) | 2.21(2.04,2.37) |
| Pakistan | 1500.83(1051.65,2101.82) | 3.66(2.56,5.12) |  | 4981.63(3638.47,6688.78) | 4.68(3.42,6.28) |  | 231.92(201.73,267.95) | 0.82(0.79,0.85) |
| Palau | 0.05(0.03,0.08) | 0.73(0.45,1.11) |  | 0.08(0.05,0.12) | 0.86(0.54,1.26) |  | 52.31(40.63,66.65) | 0.53(0.51,0.55) |
| Palestine | 108.19(78.08,150.05) | 15.28(11.03,21.19) |  | 630.60(484.23,803.92) | 27.45(21.08,34.99) |  | 482.85(340.78,650.08) | 2.51(2.28,2.74) |
| Panama | 48.49(41.07,56.94) | 4.58(3.88,5.37) |  | 183.92(147.66,224.49) | 9.01(7.23,11.00) |  | 279.32(213.29,356.28) | 2.31(1.93,2.70) |
| Papua New Guinea | 10.52(6.36,16.00) | 0.63(0.38,0.95) |  | 31.15(19.13,47.66) | 0.66(0.40,1.01) |  | 196.13(180.94,213.78) | 0.11(0.05,0.17) |
| Paraguay | 90.12(67.24,121.49) | 5.60(4.17,7.54) |  | 279.15(211.32,367.98) | 7.96(6.02,10.49) |  | 209.74(160.34,283.23) | 0.91(0.71,1.12) |
| Peru | 316.92(242.80,412.13) | 3.52(2.69,4.57) |  | 1129.66(835.49,1505.69) | 6.22(4.60,8.29) |  | 256.45(181.85,365.28) | 2.23(2.05,2.41) |
| Philippines | 648.38(501.37,817.10) | 2.48(1.91,3.12) |  | 1598.37(1304.96,1948.78) | 2.93(2.39,3.57) |  | 146.52(104.52,217.07) | 0.33(0.19,0.46) |
| Poland | 16739.79(15373.79,18245.68) | 92.91(85.33,101.27) |  | 9520.57(8039.17,11016.70) | 51.67(43.63,59.79) |  | -43.13(-49.10,-37.28) | -2.16(-2.28,-2.04) |
| Portugal | 1190.27(988.34,1420.54) | 25.23(20.95,30.11) |  | 1331.03(1140.03,1570.27) | 27.73(23.75,32.71) |  | 11.83(-2.53,27.92) | 0.54(0.46,0.62) |
| Puerto Rico | 312.89(283.22,349.09) | 18.70(16.92,20.86) |  | 337.64(275.51,414.16) | 22.91(18.70,28.11) |  | 7.91(-11.43,31.72) | 0.70(0.50,0.91) |
| Qatar | 35.19(22.37,52.20) | 12.78(8.12,18.95) |  | 522.00(356.28,707.83) | 23.44(16.00,31.79) |  | 1383.18(1070.46,1817.47) | 0.74(0.34,1.15) |
| Republic of Korea | 1156.68(903.64,1522.19) | 4.96(3.87,6.52) |  | 1374.79(1013.60,1826.91) | 5.20(3.83,6.90) |  | 18.86(7.31,31.05) | 0.23(0.15,0.32) |
| Republic of Moldova | 350.44(312.39,391.12) | 16.73(14.92,18.68) |  | 223.88(185.55,264.89) | 12.16(10.08,14.39) |  | -36.12(-46.41,-25.90) | -1.91(-2.21,-1.61) |
| Romania | 4729.83(4205.98,5251.53) | 43.50(38.68,48.30) |  | 2253.22(1848.32,2632.45) | 25.34(20.79,29.60) |  | -52.36(-60.33,-44.70) | -2.26(-2.46,-2.07) |
| Russian Federation | 34404.86(31818.01,37758.49) | 46.34(42.86,50.86) |  | 23244.80(20447.75,26142.68) | 33.90(29.83,38.13) |  | -32.44(-38.58,-25.71) | -2.18(-2.60,-1.76) |
| Rwanda | 51.58(37.64,71.65) | 2.00(1.46,2.77) |  | 132.81(91.45,186.05) | 2.30(1.58,3.22) |  | 157.48(114.00,221.02) | 0.15(-0.01,0.31) |
| Saint Kitts and Nevis | 4.37(3.90,4.87) | 26.15(23.33,29.13) |  | 8.25(6.63,10.18) | 26.03(20.91,32.11) |  | 88.92(50.29,133.42) | 0.12(-0.21,0.44) |
| Saint Lucia | 5.68(5.06,6.41) | 10.30(9.17,11.63) |  | 14.39(11.78,17.29) | 15.39(12.61,18.50) |  | 153.21(112.03,200.34) | 1.66(1.47,1.86) |
| Saint Vincent and the Grenadines | 2.94(2.43,3.44) | 6.72(5.55,7.86) |  | 6.31(5.15,7.59) | 11.48(9.38,13.82) |  | 114.64(81.89,155.00) | 1.73(1.54,1.92) |
| Samoa | 0.51(0.31,0.76) | 0.81(0.50,1.20) |  | 0.80(0.49,1.19) | 0.90(0.56,1.35) |  | 55.64(47.75,63.33) | 0.36(0.31,0.41) |
| San Marino | 1.63(1.03,2.36) | 13.68(8.65,19.87) |  | 2.43(1.60,3.51) | 16.56(10.91,23.93) |  | 49.27(16.74,89.37) | 0.71(0.67,0.74) |
| Sao Tome and Principe | 1.36(0.79,2.41) | 3.37(1.95,5.98) |  | 4.78(2.64,8.84) | 4.95(2.74,9.16) |  | 252.53(45.47,713.31) | 1.14(0.77,1.51) |
| Saudi Arabia | 575.63(399.42,800.31) | 8.52(5.91,11.85) |  | 4015.36(2931.89,5398.97) | 16.35(11.94,21.98) |  | 597.56(456.37,797.86) | 2.32(2.22,2.41) |
| Senegal | 315.57(121.20,753.77) | 11.88(4.56,28.37) |  | 1110.62(449.42,2699.63) | 17.21(6.96,41.83) |  | 251.94(2.31,915.65) | 1.30(1.16,1.45) |
| Serbia | 2689.23(1989.12,3760.27) | 57.85(42.79,80.89) |  | 2519.94(1833.96,3305.54) | 59.43(43.25,77.95) |  | -6.30(-37.03,32.69) | -0.03(-0.18,0.11) |
| Seychelles | 0.38(0.26,0.54) | 1.20(0.82,1.70) |  | 1.16(0.82,1.58) | 2.15(1.52,2.91) |  | 202.30(150.07,280.31) | 2.04(1.67,2.40) |
| Sierra Leone | 116.52(52.01,287.01) | 7.22(3.22,17.78) |  | 515.83(211.68,1161.61) | 13.89(5.70,31.29) |  | 342.69(32.74,1285.78) | 2.49(2.31,2.67) |
| Singapore | 39.39(29.28,52.73) | 2.23(1.66,2.99) |  | 73.80(52.64,102.62) | 2.33(1.66,3.24) |  | 87.37(73.28,100.11) | 0.06(-0.07,0.19) |
| Slovakia | 969.34(736.44,1279.10) | 38.92(29.57,51.36) |  | 988.53(744.00,1358.78) | 37.05(27.89,50.93) |  | 1.98(-31.65,50.82) | -0.30(-0.41,-0.19) |
| Slovenia | 723.47(648.77,804.20) | 73.37(65.80,81.56) |  | 401.66(325.90,480.53) | 43.07(34.94,51.52) |  | -44.48(-53.33,-34.83) | -1.97(-2.09,-1.85) |
| Solomon Islands | 0.99(0.61,1.49) | 0.81(0.50,1.22) |  | 2.83(1.77,4.10) | 0.95(0.59,1.37) |  | 186.07(171.56,208.49) | 0.63(0.59,0.67) |
| Somalia | 55.02(36.71,80.85) | 1.93(1.29,2.84) |  | 148.63(99.77,214.26) | 1.89(1.27,2.72) |  | 170.17(140.92,206.95) | -0.29(-0.46,-0.13) |
| South Africa | 1013.71(822.13,1266.11) | 6.33(5.13,7.91) |  | 2063.53(1638.20,2539.95) | 7.10(5.64,8.74) |  | 103.56(70.78,135.29) | 0.14(0.01,0.27) |
| South Sudan | 40.40(26.81,58.44) | 1.86(1.23,2.69) |  | 83.55(55.45,121.49) | 2.35(1.56,3.41) |  | 106.81(80.47,156.21) | 0.75(0.58,0.93) |
| Spain | 5565.95(4487.49,6720.65) | 30.79(24.83,37.18) |  | 9253.56(7172.02,11547.89) | 43.51(33.72,54.30) |  | 66.25(42.67,93.50) | 1.04(0.95,1.13) |
| Sri Lanka | 108.91(79.45,148.78) | 1.34(0.98,1.83) |  | 160.60(117.52,219.87) | 1.52(1.11,2.08) |  | 47.46(24.71,75.61) | 0.22(0.09,0.35) |
| Sudan | 667.79(462.80,910.56) | 8.92(6.18,12.16) |  | 3061.66(2221.88,4052.78) | 16.17(11.74,21.41) |  | 358.48(249.54,515.89) | 2.02(1.98,2.06) |
| Suriname | 10.51(8.02,12.99) | 6.10(4.66,7.54) |  | 26.63(18.71,36.08) | 9.59(6.74,12.99) |  | 153.36(82.14,260.62) | 1.77(1.61,1.94) |
| Sweden | 2992.03(2412.96,3662.42) | 72.86(58.76,89.18) |  | 3826.40(2952.63,4792.69) | 83.33(64.30,104.38) |  | 27.89(14.58,39.40) | 0.41(0.32,0.50) |
| Switzerland | 2568.06(2149.26,3034.85) | 71.33(59.70,84.30) |  | 2863.97(2362.27,3412.90) | 67.72(55.85,80.69) |  | 11.52(-3.31,25.94) | -0.06(-0.19,0.08) |
| Syrian Arab Republic | 545.95(391.68,731.72) | 12.18(8.74,16.32) |  | 1363.69(957.37,1857.60) | 21.90(15.37,29.83) |  | 149.78(93.72,221.85) | 2.02(1.74,2.30) |
| Taiwan (Province of China) | 122.44(88.46,164.19) | 1.19(0.86,1.59) |  | 411.02(298.72,527.21) | 3.41(2.48,4.37) |  | 235.70(194.84,304.62) | 1.60(0.83,2.38) |
| Tajikistan | 110.90(80.06,148.60) | 5.47(3.95,7.33) |  | 278.92(196.80,389.61) | 5.96(4.21,8.33) |  | 151.50(97.28,221.68) | 0.41(0.16,0.65) |
| Thailand | 332.54(222.70,469.72) | 1.18(0.79,1.67) |  | 563.64(408.72,763.18) | 1.68(1.22,2.27) |  | 69.49(48.19,102.77) | 1.11(1.05,1.17) |
| Timor-Leste | 2.80(1.87,4.21) | 0.85(0.57,1.27) |  | 6.02(4.15,8.52) | 1.06(0.73,1.49) |  | 114.75(88.50,164.82) | 0.65(0.56,0.74) |
| Togo | 155.66(70.57,323.48) | 11.98(5.43,24.90) |  | 528.01(224.97,1122.23) | 14.79(6.30,31.43) |  | 239.21(10.63,798.11) | 0.77(0.66,0.88) |
| Tokelau | 0.00(0.00,0.01) | 0.79(0.48,1.18) |  | 0.01(0.00,0.01) | 0.86(0.53,1.26) |  | 10.78(5.12,15.87) | 0.26(0.20,0.31) |
| Tonga | 0.34(0.21,0.52) | 0.96(0.59,1.44) |  | 0.46(0.29,0.67) | 1.05(0.66,1.55) |  | 32.40(27.06,39.47) | 0.33(0.28,0.38) |
| Trinidad and Tobago | 44.82(39.01,51.46) | 8.18(7.12,9.39) |  | 101.22(78.98,128.68) | 14.87(11.60,18.90) |  | 125.86(76.82,186.43) | 1.92(1.82,2.03) |
| Tunisia | 538.57(382.28,743.11) | 15.44(10.96,21.30) |  | 2038.07(1485.25,2712.41) | 34.43(25.09,45.82) |  | 278.43(193.19,391.66) | 2.64(2.50,2.78) |
| Turkey | 5603.68(4261.55,7505.95) | 22.58(17.17,30.25) |  | 11663.02(9098.86,14388.36) | 27.52(21.47,33.95) |  | 108.13(60.39,165.77) | 0.77(0.70,0.83) |
| Turkmenistan | 208.41(169.13,249.65) | 13.99(11.36,16.76) |  | 370.81(253.66,486.79) | 14.89(10.19,19.55) |  | 77.93(25.79,131.06) | 0.53(0.36,0.69) |
| Tuvalu | 0.03(0.02,0.05) | 0.75(0.46,1.13) |  | 0.04(0.03,0.06) | 0.77(0.48,1.13) |  | 41.35(35.28,48.97) | -0.06(-0.11,-0.02) |
| Uganda | 93.92(64.29,135.85) | 1.58(1.08,2.28) |  | 320.64(218.22,452.93) | 2.00(1.36,2.83) |  | 241.38(194.48,323.05) | 0.73(0.67,0.79) |
| Ukraine | 15215.04(13876.08,16772.37) | 60.39(55.08,66.57) |  | 9695.00(7201.59,12398.09) | 46.05(34.20,58.89) |  | -36.28(-51.95,-18.67) | -2.15(-2.66,-1.64) |
| United Arab Emirates | 109.73(73.20,155.07) | 9.91(6.61,14.00) |  | 999.98(685.11,1369.32) | 13.85(9.49,18.97) |  | 811.34(612.68,1069.34) | 1.01(0.68,1.33) |
| United Kingdom | 20378.73(17801.16,23138.94) | 73.80(64.47,83.80) |  | 29759.27(25188.22,34337.28) | 95.49(80.83,110.18) |  | 46.03(39.09,52.43) | 1.06(0.93,1.20) |
| United Republic of Tanzania | 187.83(127.19,265.99) | 2.06(1.40,2.92) |  | 589.21(408.52,827.24) | 2.51(1.74,3.53) |  | 213.70(162.71,298.71) | 0.62(0.55,0.68) |
| United States of America | 73482.32(60273.29,88330.01) | 57.59(47.24,69.23) |  | 91780.46(75626.43,109300.06) | 60.75(50.06,72.34) |  | 24.90(16.72,34.19) | 0.09(-0.18,0.35) |
| United States Virgin Islands | 6.90(4.76,9.46) | 13.70(9.45,18.77) |  | 5.72(3.82,8.43) | 16.12(10.77,23.76) |  | -17.14(-47.37,28.82) | 0.91(0.72,1.11) |
| Uruguay | 388.71(336.17,445.84) | 28.01(24.23,32.13) |  | 397.97(333.84,473.75) | 24.90(20.89,29.65) |  | 2.38(-10.47,16.97) | -0.49(-0.62,-0.37) |
| Uzbekistan | 615.28(455.32,817.99) | 7.39(5.47,9.82) |  | 1269.38(925.01,1703.84) | 7.48(5.45,10.04) |  | 106.31(74.69,140.10) | 0.49(0.26,0.73) |
| Vanuatu | 0.50(0.30,0.75) | 0.84(0.51,1.27) |  | 1.24(0.76,1.85) | 0.91(0.55,1.35) |  | 150.31(132.61,165.47) | 0.22(0.19,0.26) |
| Venezuela (Bolivarian Republic of) | 539.56(476.49,617.03) | 6.61(5.84,7.56) |  | 2183.95(1705.17,2773.74) | 17.27(13.49,21.94) |  | 304.77(220.17,414.84) | 3.01(2.72,3.29) |
| Viet Nam | 329.30(223.54,480.47) | 1.19(0.81,1.74) |  | 904.81(632.27,1253.63) | 1.77(1.24,2.45) |  | 174.77(132.11,240.87) | 1.19(1.13,1.24) |
| Yemen | 365.95(250.81,527.62) | 8.27(5.67,11.93) |  | 1961.74(1374.94,2761.51) | 14.09(9.88,19.83) |  | 436.07(335.77,601.01) | 1.77(1.74,1.81) |
| Zambia | 69.84(48.30,101.18) | 2.52(1.74,3.65) |  | 251.10(175.60,349.40) | 3.15(2.20,4.38) |  | 259.54(178.54,360.85) | 0.66(0.55,0.77) |
| Zimbabwe | 71.48(45.13,103.56) | 1.95(1.23,2.83) |  | 143.66(91.69,200.59) | 2.21(1.41,3.08) |  | 100.99(88.30,113.29) | 0.33(0.21,0.45) |
